# CAMI-DM: Development and validation of a multi-algorithm model for in-hospital mortality risk prediction in diabetic patients with acute myocardial infarction — The China acute myocardial infarction registry

**DOI:** 10.1371/journal.pdig.0001529

**Published:** 2026-08-07

**Authors:** Zuoxiang Wang, Zheng Yin, Junxing Lv, Sheng Zhao, Zhengqing Ba, Jingang Yang, Haiyan Xu, Xiaojin Gao, Yongjian Wu, Yuejin Yang

**Affiliations:** Department of Cardiology, Fuwai Hospital, National Center for Cardiovascular Diseases, Chinese Academy of Medical Sciences and Peking Union Medical College, Beijing, China; Shanghai United Imaging Intelligence Co Ltd, CHINA

## Abstract

Patients with diabetes constitute a substantial proportion of those with acute myocardial infarction (AMI) and exhibit distinct pathophysiological characteristics. However, existing guideline-recommended traditional and generic risk prediction models show limited performance in this specific population. Based on the China Acute Myocardial Infarction (CAMI) registry, 6,091 diabetic patients with AMI were enrolled and randomly divided into training and test sets (8:2). A comprehensive set of 62 multidimensional candidate features was extracted, and three feature selection strategies were applied to develop 12 machine learning models across six algorithm categories. All models underwent hyperparameter tuning via five-fold cross-validation in the training set, with the optimal combination selected according to the area under the receiver operating characteristic curve (AUROC). Two post-hoc ensemble strategies—stacking and probability averaging—were then employed to explore various combinations of top-performing models from different algorithm categories. Across six algorithm categories, the predictive models developed using 11 features selected by Elastic Net had the optimal performance. After evaluating various fusion strategies, the ensembled GLM + TabNet model was ultimately selected as the CAMI-DM model 2.0, achieving an AUROC of 0.875 in the test set. To balance the predictive performance and model simplicity, the CAMI-DM model 1.0 adopted a linear framework and was developed using five features from consensus feature selection Strategy, with an AUROC of 0.821 in the test set. Comparative analyses revealed that the CAMI-DM 2.0 outperformed CAMI-DM 1.0 in terms of discrimination, accuracy, calibration, and clinical net benefit. Furthermore, CAMI-DM 1.0, with its simplified structure, still outperformed the GRACE score in the overall predictive performance and generalizability. This study focused on the specific population of AMI patients with diabetes, and for the first time developed two dedicated models to predict in-hospital mortality risk.

## Introduction

Acute myocardial infarction (AMI), the most severe clinical manifestation of coronary artery disease, remains one of the leading causes of mortality worldwide and impose a substantial burden on healthcare systems [1,2]. Epidemiological data reveal a concerning upward trend in AMI mortality in China, with both urban and rural areas exceeding 60 deaths per 100,000 population in 2020 [3]. Similarly, in the United States, approximately 750,000 individuals experience either the first or recurrent AMI annually [4]. Notably, patients with diabetes mellitus (DM) represent a distinct subgroup within the myocardial infarction population, accounting for 20%-30% of all AMI cases [5,6]. Previous studies indicated that due to multiple underlying mechanisms, including more complex coronary artery lesions, endothelial dysfunction, prothrombotic state, and greater comorbidity burden, patients with diabetes experience significantly worse short- and long-term outcomes compared with those without diabetes [5]. Specifically, the in-hospital mortality rate following AMI is 1.5 to 2 times higher in patients with diabetes than non-diabetic individuals [7,8]. Although advances in revascularization techniques have substantially improved outcomes in AMI patients overall, those with DM have disproportionately high risks, with the reinfarction rate exceeding 40% and long-term mortality remaining high [1,7]. Consequently, the clinical management of AMI in diabetic patients remains particularly challenging, underscoring the critical need for accurate risk stratification and prognostic evaluation to identify high-risk inpatients and guide timely, tailored treatment strategies. Current guidelines predominantly recommend traditional risk prediction models, exemplified by the GRACE score, as standardized tools for assessing in-hospital mortality risk in patients with ACS [9]. However, the application of these models in contemporary clinical practice presents several notable limitations. First, most are based on conventional logistic regression methods, which may oversimplify the complex, often non-linear relationships between predictors and outcomes. Second, these models were developed in earlier periods. Nevertheless, patient characteristics and treatment strategies have changed dramatically in decades [10]. More importantly, as models designed for the broader ACS population, they often fail to adequately account for the unique clinical characteristics of patients with DM or incorporate metabolically relevant prognostic variables, thereby limiting their predictive performance in this high-risk subpopulation.

Recent years have witnessed rapid growth in the application of machine learning (ML) techniques for cardiovascular risk prediction. These approaches have been demonstrated to possess superior performance to conventional models across multiple clinical scenarios, owing to their capacity for processing high-dimensional data and capturing complex nonlinear relationships [11,12]. Concurrently, emerging composite metabolic indicators, such as the triglyceride-glucose index (TyG) and the stress hyperglycemia ratio (SHR), have provided new perspectives for risk prediction beyond conventional metabolic markers. These indicators have been shown to be closely associated with outcomes in patients with AMI, with particularly strong predictive values observed in those with diabetes. For example, compared with the admission glucose levels, SHR has exhibited superior accuracy in predicting in-hospital mortality [13–15]. Building upon these foundations, the purpose of the present study is to develop specialized risk prediction tools for the diabetic AMI subpopulation by applying ML algorithms, leveraging representative multicenter real-world data from China, and incorporating diabetes-specific metabolic indicators. This modelling strategy may improve risk stratification while aligning with current guideline recommendations that emphasize high-risk subpopulations and individualized assessment. [16]. To accommodate different clinical application scenarios, this study plans to develop two distinct models: CAMI-DM 1.0, a simplified model intended for rapid initial screening and bedside evaluation, and CAMI-DM 2.0, a more sophisticated model designed to provide higher predictive accuracy for comprehensive risk assessment supported by electronic health systems.

## Methods

### Data source and study population

This study utilized data from the China Acute Myocardial Infarction (CAMI) registry, a prospective, nationwide, multicenter observational study conducted across tertiary hospitals in all provinces and municipalities of mainland China (excluding Hong Kong and Macau). The registry included 108 participating hospitals, comprising 31 provincial-level, 45 municipal-level, and 32 county-level institutions, reflecting the diversity and hierarchical nature of the healthcare system in China. The study design has been described in detail in previous publications [17]. This project was approved by the Institutional Review Board of the central ethics committee of Fuwai Hospital, National Center for Cardiovascular Diseases of China (No. 2012–431). The study was registered at ClinicalTrials.gov (Identifier: NCT01874691). All procedures were conducted in strict accordance with the ethical principles of the Declaration of Helsinki, and written informed consent was obtained from eligible participants prior to registry participation.

The CAMI registry was conducted in two phases. Phase I (January 2013 to September 2014) enrolled patients with a diagnosis of ST-segment elevation myocardial infarction (STEMI) or non–ST-segment elevation myocardial infarction (NSTEMI) who were admitted within 7 days of symptom onset. In Phase II (September 2014 to January 2016), the inclusion criteria were further restricted to patients hospitalized within 3 days of symptom onset. During hospitalization, all patients received optimal medical therapy and/or coronary revascularization in accordance with contemporary national and international guidelines, the clinical judgment of the attending cardiologists, and individual patient preferences. Data were collected using standardized definitions and methodologies, and submitted through a secure, password-protected electronic data capture system to ensure data integrity and minimize information bias (S1 File, S1 Table).

**Fig 1:**
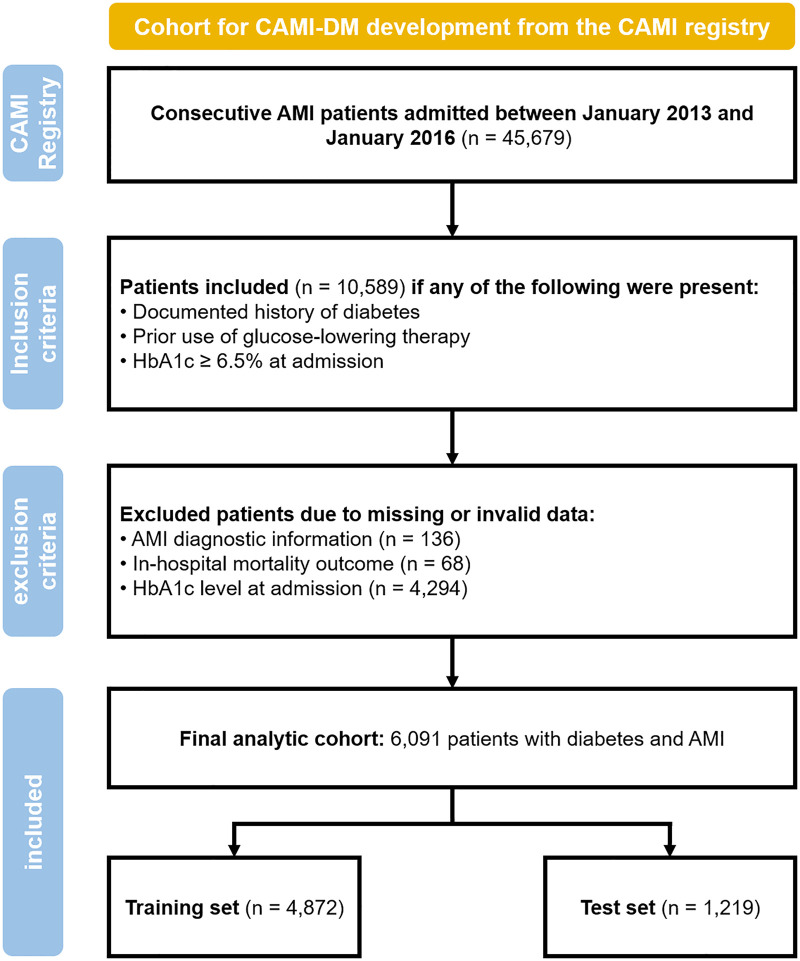
Flowchart of study cohort selection. Flowchart illustrating the inclusion and exclusion criteria, and the allocation of patients into training and test sets (8:2) for the development of the CAMI-DM model.

The present analysis was based on a total of 45,679 patients enrolled in the CAMI registry. Diabetes mellitus was defined as a documented history of diabetes, prior use of glucose-lowering therapy before admission, or a glycated haemoglobin (HbA1c) level ≥6.5% at admission. After applying rigorous inclusion and exclusion criteria, a total of 6,091 patients with diabetes were included in the primary analysis cohort (Fig 1).

### Data preprocessing and feature selection

A total of 60 candidate features were extracted from the electronic health record system through a standardized process, covering ten clinical dimensions: demographics, physical examination, medical history, echocardiographic parameters, laboratory data, electrocardiographic findings, diabetes-related variables, MI-related variables, medication use, and emergency reperfusion therapy (S2 File, S2 Table). The selection of candidate features was primarily guided by their availability and data quality within the CAMI registry, complemented by evidence from prior literature and clinical judgment. In addition, two composite indices previously demonstrated to have prognostic value in patients with myocardial infarction were calculated and included: the TyG index, defined as Ln [Triglycerides (mg/dL) × FPG (mg/dL) / 2], and the SHR, calculated as ABG (mmol/L) / (1.59 × HbA1c [%] – 2.59) [18,19].

The extent of missing data for all candidate features is presented in S1 Fig. Features with more than 20% missingness were excluded to ensure analytical quality and model stability. After splitting the dataset into training and test sets, missing values in the remaining features were imputed independently within each set using random forest-based imputation method. The primary outcome of interest was in-hospital mortality (survival vs. death). For feature selection, the univariate analysis was first conducted to exclude variables not significantly associated with in-hospital mortality (p > 0.1). Following this preliminary screening, three distinct feature selection strategies were applied independently for model construction. (1) Elastic Net Regularization: A series of 10-fold cross-validations was performed across a range of α values (representing different balances of L1 and L2 penalties). The combination of α and λ yielding the lowest mean cross-validation error was selected as the optimal hyperparameter set. Features with non-zero coefficients under the λ.1se criterion were then selected for model construction using the optimal parameters. (2) Recursive Feature Elimination (RFE): Feature importance was assessed using a random forest algorithm, followed by 5-fold cross-validation to iteratively eliminate the least important features. The number of features corresponding to the lowest cross-validation error was identified as optimal, and the top-ranked variables based on mean decrease in Gini index were retained. (3) Consensus Feature Selection: To ensure feature stability, parsimony, and predictive contribution, the features selected by Elastic Net and RFE were compared, and the overlapping features were used as the input set for model development.

### Model development

This study aimed to develop two models for predicting in-hospital mortality in AMI patients with diabetes: a simplified model suitable for clinical implementation (CAMI-DM 1.0), and a more complex model with higher predictive accuracy (CAMI-DM 2.0). First, based on three feature selection strategies, twelve commonly used supervised machine learning algorithms were developed, covering six major algorithm categories: (1) Tree-based Ensemble Models: CatBoost, XGBoost, Light Gradient Boosting Machine (LightGBM), Gradient Boosting Machine (GBM), Random Forest (RF), and Decision Tree; (2) Linear Models: Generalized Linear Model (GLM); (3) Probabilistic Models: Naive Bayes; (4) Distance-based Models: K-Nearest Neighbors Classifier (KNN); (5) Kernel-based Models: Support Vector Machine (SVM); (6) Deep Learning (DL) Models: Deep Neural Networks (DNN) and TabNet. These algorithms were selected based on their representativeness, widespread use, and methodological maturity, and to reflect major methodological paradigms with varying complexity and inductive biases, rather than to prespecify superiority of any single model class, thereby enabling a more comprehensive and interpretable comparison.

A total of 6,091 patients were randomly divided into training and test sets in an 8:2 ratio (Python, random seed: 123). In the training set, each model underwent hyperparameter tuning using either Grid Search or Optuna Bayesian optimization. During the tuning process, class weight adjustment was also included in the hyperparameter search space to mitigate the impact of class imbalance at the model level. The optimal parameter combination was selected based on AUC performance in five-fold cross-validation, and the final model was then trained using the optimal parameters and evaluated on the test set to assess generalizability. After completing the training of individual models, a post-hoc ensemble strategy was applied to further improve overall performance and stability. The best-performing model in each algorithm class was selected from the training set and used to build ensemble models through two strategies: (1) Probability averaging: the mean of predicted probabilities from the selected base models was calculated as the final prediction; (2) Stacking: predicted probabilities of multiple base models were used as input features to train a logistic regression meta-learner, and ensemble performance was subsequently evaluated in the test set.

In addition, during model analysis, we observed that linear frameworks showed stable and accurate performance, especially in settings with fewer features and lower dimensionality, and in some cases outperformed complex models while maintaining good interpretability. Therefore, a simplified prognostic model for initial rapid risk assessment, was developed using a logistic regression model and then converted into a clinically applicable scoring system. The scoring process was based on standardized transformation of regression coefficients and the intercept into integer values. According to the frequency distribution in the training set, patients were categorized into low-, intermediate-, and high-risk groups to enable rapid stratification of in-hospital mortality risk and to assist in bedside therapeutic decision-making.

### Model evaluation and interpretation

Model performance was evaluated using a range of metrics derived from the confusion matrix. The confusion matrix consists of four components: true positives (TP), true negatives (TN), false positives (FP), and false negatives (FN). The evaluated metrics included accuracy, sensitivity, specificity, positive predictive value (PPV), negative predictive value (NPV), the area under the receiver operating characteristic curve (AUROC) and area under the precision-recall curve (AUPRC). Specifically, the metrics were calculated as follows: Accuracy = (TP + TN) / (TP + TN + FP + FN), Sensitivity = TP / (TP + FN), Specificity = TN / (TN + FP), PPV = TP / (TP + FP), NPV = TN / (TN + FN). AUROC was used as the primary indicator of discrimination performance, and its 95% confidence interval was estimated using bootstrap resampling to assess statistical stability.

To enhance interpretability, the Shapley Additive explanations (SHAP) method was applied both to the CAMI-DM 2.0 model and its component sub-models. SHAP is based on the Shapley value theory from cooperative game theory and is used to quantify the marginal contribution of each input variable to individual predictions, offering strong theoretical rigor and broad model applicability. In this study, the KernelExplainer was adopted as the explainer, and SHAP values were calculated on the entire test set. The SHAP analysis results were visualized in the form of global feature importance bar charts and decision plots, which not only presented the model’s overall dependence on different features but also allowed tracing the prediction logic at the individual level, thereby improving clinical transparency and interpretability.

For the finalized CAMI-DM 1.0 and CAMI-DM 2.0 models, along with the reference Global Registry of Acute Coronary Events (GRACE) score as a comparator, we conducted comprehensive performance comparisons using multidimensional statistical approaches. The DeLong’s test was used to assess differences in AUROC between models, and the integrated discrimination improvement (IDI) and net reclassification improvement indices (NRI) were calculated to quantify improvements in the reclassification performance. Calibration was assessed using calibration plots and the Hosmer–Lemeshow goodness-of-fit test. Finally, to assess clinical utility, decision curve analysis (DCA) was performed to quantify net benefit across various risk threshold probabilities.

Considering the potential residual heterogeneity within the study population, exploratory subgroup analyses were conducted to assess model performance in internally heterogeneous patient groups. Specifically, Patients were stratified separately according to diabetes status (newly recognized diabetes vs. previous diabetes) and enrolment phase (Phase I vs. Phase II). Within each subgroup, the predictive performance of CAMI-DM 2.0, CAMI-DM 1.0, and the GRACE score was evaluated and compared.

### Statistical analysis

Baseline characteristics of the study population were described from two aspects. First, patients were divided into in-hospital death and survival groups based on the outcome (Table 1). Second, population characteristics were compared between the training and test sets used for model development (S3 Table). Continuous variables were summarized as means (±SD) or medians (IQR) according to the data distribution, and categorical variables were expressed as frequencies (percentages). Group differences were assessed using the Wilcoxon rank-sum test or Pearson’s chi-squared test, as appropriate for variable type and distribution. All statistical tests were two-sided, with statistical significance defined at p < 0.05. Statistical analyses were performed using R software (version 4.2.2, R Foundation for Statistical Computing) and Python (version 3.12; Python Software Foundation).

**Table 1:** Baseline characteristics.

Characteristic	In-hospital survivors N = 5,798	In-hospital deaths N = 293	p-value
**Demographics**			
**Age (years), Mean (SD)**	63 (12)	71 (10)	<0.001^1^
**Sex, Male, n (%)**	4,120 (71.1%)	142 (48.5%)	<0.001^2^
**BMI (kg/m** ^**2**^ **), Median (IQR)**	24.5 (22.7, 26.6)	23.8 (21.6, 25.5)	<0.001^1^
**Smoking status, Current, n (%)**	2,292 (39.8%)	64 (21.8%)	<0.001^2^
**Medical history**			
**Hypertension, n (%)**	3,534 (61.8%)	199 (69.1%)	0.013^2^
**Dyslipidemia, n (%)**	4,188 (81.5%)	178 (72.7%)	<0.001^2^
**Previous angina, n (%)**	1,652 (36.0%)	72 (31.6%)	0.2^2^
**Previous MI (>1 month), n (%)**	541 (11.9%)	25.0 (10.8%)	0.6^2^
**Previous HF, n (%)**	166 (3.0%)	25 (9.2%)	<0.001^2^
**Family history of premature CAD, n (%)**	242 (5.0%)	7 (3.0%)	0.2^2^
**Previous PCI, n (%)**	366 (7.8%)	6 (2.5%)	0.002^2^
**Previous CABG, n (%)**	45 (0.9%)	0 (0.0%)	0.2^3^
**Previous stroke, n (%)**	566 (10.2%)	58 (21.0%)	<0.001^2^
**Previous COPD, n (%)**	99 (1.8%)	7 (2.5%)	0.4^2^
**Previous PAD, n (%)**	71 (1.3%)	3 (1.1%)	>0.9^3^
**Previous digestive ulcer, n (%)**	112 (3.2%)	6 (3.3%)	>0.9^2^
**Previous Bleed, n (%)**	58 (1.7%)	4 (2.2%)	0.5^3^
**Previous Cancer, n (%)**	87 (1.6%)	7 (2.5%)	0.2^3^
**Echocardiographic parameters**			
**LVEDd (mm), Median (IQR)**	50 (46.0, 54.0)	51 (47.0, 55.0)	0.059^1^
**EF (%), Mean (SD)**	53 (10)	45 (12)	<0.001^1^
**Laboratory data**			
**SCR (μmol/L), Median (IQR)**	75 (62, 92)	96 (75, 140)	<0.001^1^
**HB (g/L), Mean (SD)**	135 (20)	124 (22)	<0.001^1^
**PLT (10** ^**9**^ **/L), Median (IQR)**	204 (168, 247)	217 (163, 267)	0.087^1^
**WBC (10** ^**9**^ **/L), Median (IQR)**	9.5 (7.5, 11.9)	11.5 (8.6, 15.1)	<0.001^1^
**K+ (mmol/L), Mean (SD)**	4.03 (0.50)	4.16 (0.69)	0.003^1^
**Na+ (mmol/L), Mean (SD)**	138.4 (4.2)	137.6 (5.1)	0.008^1^
**Cl- (mmol/L), Mean (SD)**	101.9 (4.7)	101.0 (5.4)	0.027^1^
**TC (mmol/L), Median (IQR)**	4.50 (3.78, 5.31)	4.43 (3.47, 5.19)	0.091^1^
**TG (mmol/L), Median (IQR)**	1.59 (1.13, 2.35)	1.37 (0.99, 2.00)	<0.001^1^
**LDL-C (mmol/L), Median (IQR)**	2.71 (2.15, 3.38)	2.65 (2.01, 3.41)	0.5^1^
**HDL-C (mmol/L), Median (IQR)**	1.00 (0.80, 1.20)	1.02 (0.84, 1.20)	0.029^1^
**Diabetes-Related variables**			
**Duration of DM, Median (IQR)**	2 (0, 8)	4 (0, 10)	0.003^1^
**DM medications (Insulin), n (%)**	1,049.0 (19.3%)	56.0 (21.2%)	0.4^2^
**Glu (mmol/L), Median (IQR)**	9.7 (7.3, 13.2)	11.8 (8.8, 17.0)	<0.001^1^
**HbA1C (%), Median (IQR)**	7.51 (6.70, 9.00)	7.50 (6.70, 9.33)	0.6^1^
**Composite indices**			
**SHR, Median (IQR)**	0.99 (0.80, 1.26)	1.24 (0.93, 1.61)	<0.001^1^
**TyG, Median (IQR)**	9.45 (8.98, 9.95)	9.53 (8.97, 10.02)	0.2^1^
**Physical Examination**			
**HR (beats/min), Mean (SD)**	80 (18)	89 (24)	<0.001^1^
**SBP (mmHg), Mean (SD)**	132 (25)	122 (27)	<0.001^1^
**SBP Range, n (%)**			<0.001^2^
90-180mmHg	5,282 (91.9%)	247 (84.9%)	
≥180mmHg	255 (4.4%)	7 (2.4%)	
≤90mmHg	210 (3.7%)	37 (12.7%)	
**DBP (mmHg), Mean (SD)**	79 (15)	74 (16)	<0.001^1^
**MAP (mmHg), Mean (SD)**	97 (17)	90 (19)	<0.001^1^
**Killip classification, n (%)**			<0.001^2^
I	4,282 (74.6%)	126 (43.4%)	
II	983 (17.1%)	66 (22.8%)	
III	307 (5.3%)	45 (15.5%)	
IV	169 (2.9%)	53 (18.3%)	
**MI-related variables**			
**MI Type, n (%)**			0.7^2^
NSTEMI	1,670 (28.8%)	88 (30.0%)	
STEMI	4,128 (71.2%)	205 (70.0%)	
**Time to hospital (>6 hours), n (%)**	2,650 (55.9%)	154 (63.6%)	0.017^2^
**MI with cardiac arrest, n (%)**	43 (0.7%)	10 (3.4%)	<0.001^3^
**Malignant arrhythmia, n (%)**	201 (3.5%)	35 (12.2%)	<0.001^2^
**Electrocardiographic findings**			
**NCLBBB, n (%)**	51 (0.9%)	8 (2.7%)	0.007^3^
**NCRBBB, n (%)**	122 (2.1%)	14 (4.8%)	0.003^2^
**ST-segment elevation, n (%)**	3,750 (65.0%)	193 (65.9%)	0.8^2^
**ST-segment depression, n (%)**	1,117 (19.4%)	79 (27.0%)	0.001^2^
**Pathological Q wave, n (%)**	1,055 (18.3%)	59 (20.1%)	0.4^2^
**AVB, n (%)**	89 (1.5%)	17 (5.9%)	<0.001^2^
**Medications**			
**Aspirin, n (%)**	5,587 (96.8%)	264 (91.0%)	<0.001^2^
**P2Y_12_ inhibitor, n (%)**	5,651 (97.9%)	274 (94.2%)	<0.001^2^
**β-blocker, n (%)**	4,064 (71.1%)	138 (48.1%)	<0.001^2^
**RAS inhibitor, n (%)**	4,196 (73.5%)	141 (49.0%)	<0.001^2^
**Statin, n (%)**	5,393 (97.5%)	243 (89.7%)	<0.001^2^
**Emergency Reperfusion Therapy, n (%)**	2,665 (46.0%)	71 (24.2%)	<0.001^2^
**Thrombolytic Therapy, n (%)**	465 (8.0%)	14 (4.8%)	0.044^2^
**PCI, n (%)**	2,272 (39.2%)	58 (19.8%)	<0.001^2^
**CABG, n (%)**	5 (0.1%)	1 (0.3%)	0.3^3^

^1^Wilcoxon rank sum test; ^2^Pearson’s Chi-squared test; ^3^Fisher’s exact test.

## Results

### Baseline characteristics

A total of 6,091 AMI patients with diabetes were included in this study. The mean age was 63 years (SD, 12 years), and 70.0% were male. Among them, 71.1% were diagnosed with ST-segment elevation myocardial infarction (STEMI). For composite metabolic indices, the median SHR was 1.00 (IQR, 0.81–1.27), and the median TyG was 9.44 (IQR, 8.97–9.95). In-hospital mortality occurred in 293 patients, accounting for 4.8% of the study population. In-hospital deaths exhibited significant differences in multiple clinical characteristics compared with survivors. Patients in the death group were older on average (71 vs. 63 years, P < 0.001) and had a markedly lower proportion of males (48.5% vs. 71.1%, P < 0.001). Physical examination findings showed that these patients presented with higher heart rates (HR) (89 vs. 80 beats/min, P < 0.001) and lower systolic blood pressure (SBP) (122 vs. 132 mmHg, P < 0.001). The Killip classification was also higher in the death group, with a significantly increased proportion of Killip class IV patients (22.5% vs. 3.8%, P < 0.001). Moreover, patients who died had lower ejection fraction (EF) (45% vs. 53%, P < 0.001), elevated serum creatinine (SCR) (96 vs. 75 μmol/L, P < 0.001), increased white blood cell counts (11.5 vs. 9.5 × 10⁹/L, P < 0.001), and higher SHR (1.24 vs. 0.99, P < 0.001), indicating a more severe degree of organ dysfunction and metabolic stress in this group. Patients were randomly assigned to a training set (n = 4,872) and a test set (n = 1,219) in an 8:2 ratio. Baseline characteristics, including demographics, physical examination, medical history, and laboratory findings, were generally well balanced between the two groups (S3 Table). Although a few variables showed P values < 0.05, overall distributions were comparable, supporting adequate between-group consistency for model development and validation.

### Feature selection

A total of 62 baseline features were initially included in the analysis. First, 9 features with more than 20% missing data were excluded (S1 Fig). Based on univariate logistics regression analysis, 13 features not significantly associated with in-hospital mortality (p > 0.1) were removed (S4 Table). In addition, to avoid information redundancy and feature nesting, either composite variables, derived variables, or their corresponding component features were selectively excluded based on both statistical considerations and clinical relevance. For example, since SHR was found to have a superior discrimination property compared with the random plasma glucose and HbA1C in the univariate analysis, SHR was retained while the random plasma glucose and HbA1C, as its underlying components, were excluded (S2 Fig). After these steps, 29 features remained for the feature selection stage. Three feature selection strategies were subsequently applied: (1) Elastic Net (S3 Fig): After parameter tuning, the optimal penalty coefficient was determined as α = 0.9, indicating a preference toward L1 regularization. Under the λ.1se penalty, a total of 11 non-zero coefficient features were selected: age, sex, ejection fraction (EF), SCR, haemoglobin (HB), SHR, HR, SBP, Killip class, malignant arrhythmia (MA), and emergency percutaneous coronary intervention (PCI). (2) RFE (S4 Fig): A five-fold cross-validation framework was used to evaluate prediction error across varying numbers of features. The lowest error was observed when seven features were retained. On the basis of importance ranking, the top seven variables selected were: SCR, SHR, age, serum potassium (K⁺), EF, HR, and body mass index (BMI). (3) Consensus Feature Selection: To simplify the model and enhance the robustness of feature selection, the intersection of features identified by the Elastic Net and RFE was taken. This yielded five consistently selected features: age, EF, SCR, SHR, and HR. All three feature sets (11-variable, 7-variable, and 5-variable subsets) were used to develop predictive models, and their performance was compared in subsequent analyses.

### CAMI-DM 2.0: Development, evaluation, and interpretation of an ensemble learning model

**Fig 2:**
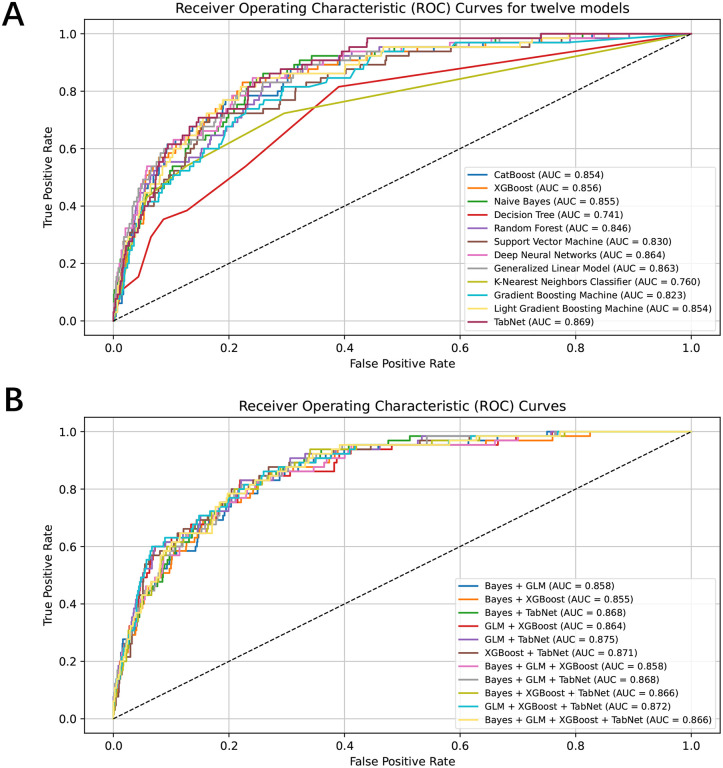
Performance comparison of machine learning models and post-hoc ensemble strategies. (A) ROC curves of twelve machine learning models (Feature Selection Strategy: Elastic Net) in the independent test set, including CatBoost, XGBoost, Naive Bayes, Decision Tree, Random Forest, Support Vector Machine, Deep Neural Networks, Generalized Linear Model, K-Nearest Neighbors, Gradient Boosting Machine, Light Gradient Boosting Machine, and TabNet. (B) ROC curves of various model combinations based on the probability averaging ensemble strategy in the independent test set.

Twelve machine learning models were developed under three distinct feature selection strategies. Comprehensive hyperparameter search and tuning were performed within the training set, and the parameter combination yielding the highest AUROC in five-fold cross-validation was selected. The final models were then evaluated on the test set (S4 Table). Regarding overall model performance, models developed under Feature Selection Strategy 1 demonstrated markedly the superior discrimination ability compared with those based on Strategies 2 and 3, with only minor performance differences observed between the latter two. Within Strategy 1, the TabNet model showed the best discriminative power, with an AUROC of 0.869 (95% CI: 0.833–0.904). Additionally, the linear-modelling framework GLM and the probabilistic-modelling category Naive Bayes model also exhibited strong predictive capabilities, with AUROCs of 0.863 (95% CI: 0.819–0.902) and 0.855 (95% CI: 0.809–0.894), respectively. Among the tree-based models, XGBoost performed best, yielding an AUROC of 0.856 (95% CI: 0.810–0.898). In Strategy 2, TabNet again ranked first with an AUROC of 0.841 (95% CI: 0.791–0.888), while in Strategy 3, GLM achieved the highest performance, with an AUROC of 0.838 (95% CI: 0.788–0.883) (S5 Fig, Fig 2).

Building upon the above modelling results, the top-performing models from each algorithm category based on training-set performance under Feature Selection Strategy 1—namely, TabNet (deep learning model), GLM (linear model), Naive Bayes (probabilistic model), and XGBoost (tree-based model)—were selected for a late-fusion ensemble modelling. Two ensemble strategies were evaluated: probability averaging and stacking. Multiple model combinations were tested, including all possible two-model, three-model, and four-model ensembles. Ensemble performance was assessed on the independent test set. In the probability averaging fusion strategy, the two-model combination of TabNet and GLM demonstrated the best performance, achieving an AUROC of 0.875 (95% CI: 0.835–0.911), outperforming all other combinations (Fig 2). In the stacking fusion strategy, with the logistic regression as the meta-learner, the TabNet + GLM combination again yielded the highest AUROC of 0.875 (95% CI: 0.836–0.911) (S6 Fig). These findings indicate a favourable trade-off between model stability and predictive performance. Given its high discrimination property, lower complexity, and superior clinical interpretability, the probability-fused model combining TabNet and GLM was ultimately selected as the final model, designated CAMI-DM 2.0, representing the prognostic tool with high accuracy and sophistication developed in this study (S7 Fig).

**Fig 3:**
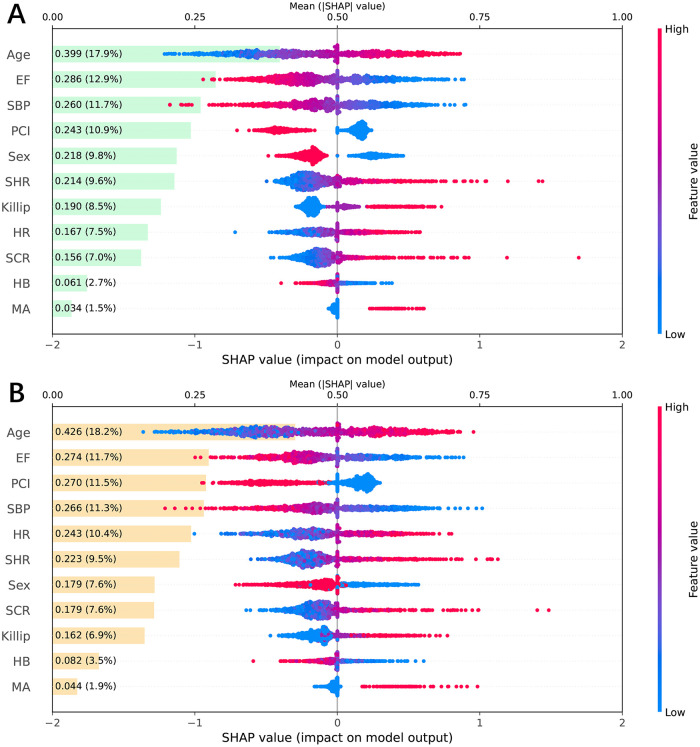
Feature importance interpretation using SHAP values. SHAP summary plot of the (A) ensemble model CAMI-DM 2.0 (combining GLM and TabNet) and (B) TabNet submodel. Each dot represents an individual prediction. Features are ranked by their mean absolute SHAP values, indicating their overall contribution to the model output. The x-axis reflects the direction and magnitude of impact on the prediction (right: positive, left: negative). The color gradient represents the original feature value (red: high, blue: low). EF, ejection fraction; SBP, systolic blood pressure; PCI, (emergency) percutaneous coronary intervention; SHR, stress hyperglycemia ratio; HR, heart rate; SCR, serum creatinine; HB, hemoglobin; MA, malignant arrhythmia.

To enhance the interpretability of the model, this study employed SHAP to analyze the importance and directional impact of each feature. Based on the SHAP summary plot of the overall ensemble model, features most strongly associated with in-hospital mortality included age, EF, SBP, emergency PCI, sex, SHR, Killip class, HR, and SCR. Higher values of age, HR, SCR, and SHR were significantly associated with an increased risk of mortality, as reflected by SHAP values predominantly distributed in the positive range. In contrast, higher EF and SBP contributed negatively to the prediction, indicating potential protective effects. Further examination of the SHAP analysis for the TabNet sub-model revealed a similar ranking of key features, demonstrating consistent and stable reliance on both structural and metabolic indicators. This consistency supports the critical role of TabNet within the ensemble architecture (Fig 3). An exploration of the prediction pathway for the CAMI-DM 2.0 model was conducted using a SHAP decision plot, which visually illustrated how different feature combinations shaped individual prediction outcomes. In the plot, each line represents a single patient, with blue lines indicating predicted survival and red lines indicating predicted mortality. The slope and inflection points of each line reflect both the direction and magnitude of each feature’s contribution, providing insight into the step-by-step reasoning behind the model’s prediction (S8 Fig).

### CAMI-DM 1.0: Development and validation of a simplified scoring model

**Fig 4:**
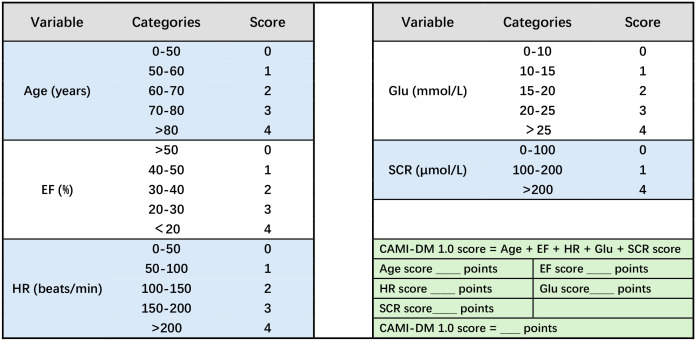
Scoring system of the CAMI-DM 1.0. The CAMI-DM 1.0 is a simplified point-based model incorporating five variables: age, ejection fraction (EF), heart rate (HR), admission blood glucose (Glu), and serum creatinine (SCR). The total score is calculated by summing the individual component scores: CAMI-DM 1.0 score = Age score + EF score + HR score + Glu score + SCR score.

**Fig 5:**
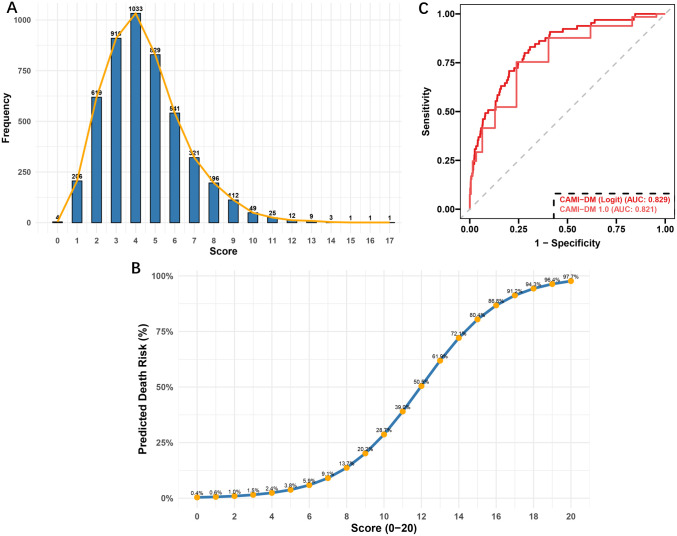
Characteristics and performance of the CAMI-DM 1.0. (A) Score distribution of CAMI-DM 1.0 in the training cohort. (B) Estimated in-hospital mortality risk corresponding to CAMI-DM 1.0 scores ranging from 0 to 20 in the training set. The predicted risk increased from 0.4% to 97.7% as the total score increased. (C) ROC curves of the CAMI-DM 1.0 model and its original logistic regression counterpart in the independent test set.

During the development of the CAMI-DM 2.0 model, we observed that the linear modelling framework performed well across all three feature selection strategies. Given that another objective of this study was to develop a prediction tool that is simple in structure, easily interpretable, and suitable for rapid bedside application, we constructed logistic regression models in the training set based on each feature selection strategy (S9 Fig). After a comprehensive evaluation of both predictive performance and model complexity, the model derived from Strategy 3—age, EF, HR, SHR, and SCR—was ultimately selected. With consideration of the need for rapid bedside calculation, the SHR variable was simplified by substituting it with the more readily available random plasma glucose measurement. Although this replacement resulted in a slight reduction in predictive accuracy, it significantly enhanced clinical practicality. Based on the logistic regression coefficients (β) and intercept, continuous predictors were converted into simplified categorical intervals to construct the CAMI-DM 1.0 scoring system. The cut-points and simplified categorical interval were determined with reference to the distribution, median value, and observed risk gradient of each feature in the training set, while also considering bedside operational convenience. For each interval, a representative value was assigned, and its difference from the baseline reference value was multiplied by the corresponding β coefficient to estimate the interval-specific contribution to in-hospital mortality risk. These values were then rounded and converted into integer point values. The CAMI-DM 1.0 score was calculated by summing the points assigned to each predictor (S10 Fig, S5 Table, Fig 4). [20]. The total score ranges from 0 to 20 and corresponds to an estimated in-hospital mortality risk between 0.4% and 97.7%. In the training cohort, patients were stratified into three risk groups—low, intermediate, and high—according to tertiles of the score derived from the scoring system (Fig 5). The low-risk group (0–3 points; first tertile) exhibited an in-hospital mortality rate of 0.75% (13/1,739), with a predicted risk range of 0.38% to 1.53%. Among patients in the intermediate-risk group (4–5 points; second tertile), the mortality rate rose to 2.36% (44/1,862), with an estimated risk range of 2.41% to 3.78%. In contrast, the high-risk group (≥6 points; third tertile) demonstrated a markedly elevated mortality rate of 13.45% (171/1,271), corresponding to a predicted risk range of 5.89% to 91.25%. Trend tests and pairwise comparisons revealed statistically significant differences among the three groups, indicating that the scoring system provides effective stratification of in-hospital mortality risk (Table 2). This model was designated as the CAMI-DM 1.0, representing a concise and practical prediction tool suitable for rapid bedside application. In the independent test cohort, the CAMI-DM 1.0 model showed an AUROC of 0.821, which was slightly lower than that of the standard logistic regression model constructed using the same five features (AUROC = 0.829) (Fig 5). This marginal reduction in performance was within an acceptable range and aligned with expectations given the simplified design of the scoring system, which nonetheless maintained strong predictive capability. The risk stratification remained robust in the test set: in-hospital mortality rates were 0.90% (4/445) in the low-risk group (0–3 points), 2.67% (12/449) in the intermediate-risk group (4–5 points), and 15.08% (49/325) in the high-risk group (≥6 points) (Table 2). Trend tests and pairwise comparisons between groups showed statistically significant differences, further supporting the generalizability and clinical applicability of the CAMI-DM 1.0 model across different datasets.

**Table 2:** Risk stratification by CAMI-DM 1.0 and corresponding In-Hospital death rates.

	Risk Group	Total Cases (n)	Deaths (n)	Observed Death Rate (%)	Predicted Risk Range (%)	Trend P-Value^1^	P-Value^2^	
**Training set**	**Low Risk (Score 0–3)**	**1,739**	**13**	**0.75%**	**0.38% - 1.53%**	**<0.001**	**——**	**——**
	**Intermediate Risk (Score 4–5)**	**1,862**	**44**	**2.36%**	**2.41% - 3.78%**		**<0.001**	**——**
	**High Risk (Score ≥6)**	**1,271**	**171**	**13.45%**	**5.89% - 91.25%**		**<0.001**	**<0.001**

**Test set**	**Low Risk (Score 0–3)**	**445**	**4**	**0.90%**	**0.38% - 1.53%**	**<0.001**	**——**	**——**
	**Intermediate Risk (Score 4–5)**	**449**	**12**	**2.67%**	**2.41% - 3.78%**		**<0.001**	**——**
	**High Risk (Score ≥6)**	**325**	**49**	**15.08%**	**5.89% - 50.48%**		**0.003**	**<0.001**

**1.Trend Test (Cochran-Armitage)**

**2. Chi-squared test: Intermediate vs Low, High vs Low, Intermediate vs High.**

**Comparative evaluation of CAMI-DM 1.0, CAMI-DM 2.0, and the GRACE score**

**Fig 6:**
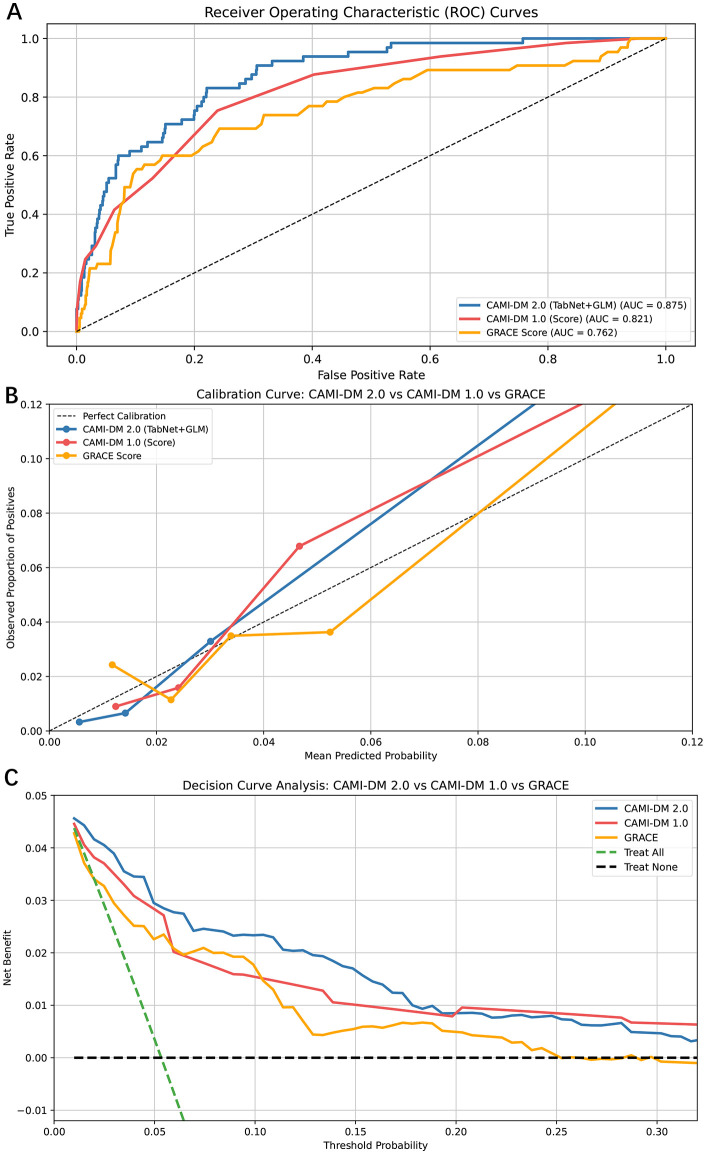
Performance comparison of CAMI-DM 2.0, CAMI-DM 1.0, and the GRACE score. (A) ROC curves, (B) calibration curves and (C) decision curve analysis comparing CAMI-DM 2.0, CAMI-DM 1.0, and the GRACE score in the independent test set.

In the independent test set, the CAMI-DM 2.0 demonstrated the highest discriminative performance, with an AUROC of 0.875 (95% CI: 0.840–0.906), significantly exceeding that of the CAMI-DM 1.0 (AUROC = 0.821, 95% CI: 0.777–0.861) and the GRACE score (AUROC = 0.762, 95% CI: 0.703–0.817) (Fig 6). Statistical comparisons using DeLong’s test confirmed that the CAMI-DM 2.0 significantly outperformed both the CAMI-DM 1.0 and GRACE in terms of AUROC (p < 0.001). The difference between CAMI-DM 1.0 and GRACE was also statistically significant (p = 0.046). Given the imbalanced outcome distribution, AUPRC was further assessed. CAMI-DM 2.0 achieved the highest AUPRC of 0.363 (95% CI: 0.270-0.460), followed by CAMI-DM 1.0 with an AUPRC of 0.283 (95% CI: 0.201-0.382) and the GRACE score with an AUPRC of 0.193 (95% CI: 0.139-0.275). Beyond AUROC and AUPRC, the CAMI-DM 2.0 also demonstrated strong and well-balanced performance across other metrics, with an accuracy of 0.782 (95% CI: 0.727-0.848), sensitivity of 0.831 (95% CI: 0.765-0.902), specificity of 0.779 (95% CI: 0.718-0.850), PPV of 0.175 (95% CI: 0.138-0.230), and NPV of 0.988 (95% CI: 0.983-0.993). These findings suggest that the model is particularly effective at ruling out low-risk patients while minimizing missed diagnoses, thereby enhancing clinical safety margins. In terms of reclassification performance, the CAMI-DM 2.0 showed significant improvements over the GRACE score, with an IDI of 0.061 (95% CI: 0.033–0.090, p < 0.001) and an NRI of 0.699 (95% CI: 0.448–0.944, p < 0.001), indicating enhanced accuracy in risk prediction and classification. Although the reclassification metrics for CAMI-DM 1.0 were slightly lower, they remained superior to those of the GRACE score (IDI = 0.046, 95% CI: 0.012–0.086, p = 0.018; NRI = 0.321, 95% CI: 0.070–0.559, p = 0.009) (Table 3). Regarding calibration, both CAMI-DM 2.0 and CAMI-DM 1.0 exhibited curves closely aligned with the ideal calibration line (Fig 6). The Hosmer–Lemeshow test yielded χ² = 10.82 (p = 0.094) for CAMI-DM 2.0 and χ² = 7.82 (p = 0.166) for CAMI-DM 1.0, indicating good model calibration with no evidence of statistical lack-of-fit. In contrast, the GRACE score demonstrated comparatively poorer calibration performance, particularly in the low-risk range. Finally, in the assessment of clinical utility, the DCA showed that CAMI-DM 2.0 provided greater net benefit across a wide range of clinically relevant threshold probabilities, and the CAMI-DM 1.0 also outperformed the GRACE score, indicating the potential utility of both models in clinical decision-making (Fig 6). In summary, the CAMI-DM 2.0 outperformed the CAMI-DM 1.0 in terms of the discriminative ability, predictive accuracy, calibration consistency, and clinical net benefit. Meanwhile, the CAMI-DM 1.0, despite its simpler structure, still demonstrated moderately superior overall performance compared to the GRACE score, with strong generalizability and practical value for bedside application.

**Table 3:** Discriminative performance and comparative evaluation of CAMI-DM Models and the GRACE Score in the Independent Test Set.

Model	AUROC (95% CI)	AUPRC (95% CI)	Accuracy (95% CI)	Sensitivity (95% CI)	Specificity (95% CI)	PPV (95% CI)	NPV (95% CI)
**CAMI-DM 2.0 (TabNet + GLM)**	**0.875 (0.840–0.906)**	**0.363 (0.270–0.460)**	**0.782 (0.727–0.848)**	**0.831 (0.765–0.902)**	**0.779 (0.718–0.850)**	**0.175 (0.138–0.230)**	**0.988 (0.983–0.993)**
**CAMI-DM 1.0 (Score)**	**0.821 (0.777–0.861)**	**0.283 (0.201–0.382)**	**0.760 (0.738–0.779)**	**0.754 (0.667–0.844)**	**0.761 (0.739–0.780)**	**0.151 (0.117–0.184)**	**0.982 (0.975–0.989)**
**GRACE Score**	**0.762 (0.703–0.817)**	**0.193 (0.139–0.275)**	**0.754 (0.683–0.854)**	**0.692 (0.605–0.788)**	**0.757 (0.679–0.865)**	**0.138 (0.104–0.223)**	**0.978 (0.971–0.986)**

**Comparison**	**DeLong p.value**	**IDI**	**IDI 95% CI**	**IDI p.value**	**NRI**	**NRI 95% CI**	**NRI p.value**
**CAMI-DM 2.0 vs CAMI-DM 1.0**	**<0.001**	**0.014**	**(-0.010, 0.039)**	**0.239**	**0.463**	**(0.215, 0.721)**	**<0.001**
**CAMI-DM 2.0 vs GRACE**	**<0.001**	**0.061**	**(0.033, 0.090)**	**<0.001**	**0.699**	**(0.448, 0.944)**	**<0.001**
**CAMI-DM 1.0 vs GRACE**	**0.046**	**0.046**	**(0.012, 0.086)**	**0.018**	**0.321**	**(0.070, 0.559)**	**0.009**

**PPV: Positive Predictive Value; NPV: Negative Predictive Value; IDI: Integrated Discrimination Improvement; NRI: Net Reclassification Improvement; 95% confidence intervals (CIs) were estimated using 1,000 bootstrap resamples.**

The results of the subgroup analyses in the test set are summarized in S6 Table. For the diabetic subgroups, CAMI-DM 2.0 consistently demonstrated the highest discriminative performance, with an AUROC of 0.873 (95% CI: 0.797–0.936) for newly recognized diabetes and 0.876 (95% CI: 0.840–0.911) for previously diagnosed diabetes. CAMI-DM 1.0 showed slightly lower performance, with an AUROC of 0.803 (95% CI: 0.714–0.881) and 0.832 (95% CI: 0.782–0.879), respectively, whereas the GRACE score exhibited the lowest performance, with an AUROC of 0.755 (95% CI: 0.650–0.848) and 0.767 (95% CI: 0.690–0.834), respectively. Similarly, when stratified by enrolment phase, CAMI-DM 2.0 achieved the highest AUROC in both Phase I (0.855, 95% CI: 0.773–0.927) and Phase II (0.880, 95% CI: 0.843–0.912). CAMI-DM 1.0 ranked next (0.823, 95% CI: 0.724–0.910; 0.815, 95% CI: 0.767–0.861), followed by the GRACE score (0.671, 95% CI: 0.531–0.799; 0.793, 95% CI: 0.734–0.854). Overall, these results indicate that the CAMI-DM models maintain generally stable discrimination across internally heterogeneous subgroups. Minor differences in model performance between subgroups were observed, yet the overall ranking and advantage remained consistent.

## Discussion

In this large, nationally representative cohort of Chinese AMI population, we developed the first large-scale risk prediction model exclusively for in-hospital mortality among individuals with DM. To meet diverse clinical needs, two differentiated models were developed: (I) the CAMI-DM 1.0, a simplified and interpretable point-based scoring model derived from five readily available variables at an early stage of hospitalization, intended for early bedside risk identification and stratification; and (II) the CAMI-DM 2.0, a high-complexity ensemble model integrating a broader range of clinical information to improve predictive accuracy and stability, optimally suited for comprehensive risk assessment after the reperfusion strategy has been determined and implemented with the availability of additional clinical data. In addition, our analysis revealed that the SHR outperformed its individual components in predicting in-hospital mortality among DM patients with AMI, suggesting the potential pivotal role of metabolic stress in shaping clinical outcomes in this population.

Over the past few decades, numerous risk prediction models have been developed for patients with AMI, primarily based on traditional statistical approaches such as the logistic regression. Among these, the GRACE score has been the most widely adopted in clinical practice due to its practicality and simplicity [21,22]. However, such conventional models are often too simplified to capture the complex relationships between clinical features and outcomes, which limit their predictive accuracy in risk stratification and clinical decision-making [11,23]. In the absence of newly identified robust predictive variables, refining heterogeneous subpopulations and improving modeling algorithms have become important strategies for enhancing predictive performance. Previous studies have attempted to develop models tailored to specific subgroups (e.g., for female patients or NSTEMI populations), and have shown that subgroup-specific models may improve predictive accuracy compared with unified models targeting general AMI populations [10,24]. The present study focuses on the diabetic population, recognizing that individuals with diabetes differ markedly from non-diabetic patients in terms of metabolic status, stress response, coronary lesion characteristics, myocardial injury mechanisms, and treatment responsiveness, exhibiting substantial clinical and physiological heterogeneity [8,25]. Accordingly, this study incorporates DM-specific variables to develop a tailored risk prediction model, aiming to enhance the accuracy of mortality risk assessment in this population. In recent years, machine learning methods have been increasingly applied in model development, owing to their ability to capture complex nonlinear relationships that traditional statistical methods often fail to identify, thereby potentially enhancing model performance [11]. However, it is important to emphasize that different algorithms have distinct strengths, and no single model is perfect or universally superior, as deep learning/machine learning methods are not necessarily better than linear frameworks in all scenarios. Model performance largely depends on the true relationship between features and outcomes, as well as the appropriateness of the feature set in terms of number and dimensionality. When the relationship between features and outcomes is approximately linear, linear models can perform comparably to, or even better than, more complex algorithms [26]. In this study, we systematically developed multiple prediction models for the AMI population with diabetes under different feature selection strategies. Notably, the linear frameworks consistently demonstrated favorable predictive performance across all feature selection approaches, with particularly outstanding results observed in Strategy 3 characterized by reduced dimensionality and lower number of features. In the final evaluation of post-hoc ensemble modelling strategies, the combination of a GLM and TabNet was identified as the optimal configuration and was adopted as the final form of CAMI-DM 2.0. This configuration reflects the presence of a certain linear relationship between the selected features and the outcome, while TabNet effectively complements the linear model by capturing additional complex, non-linear associations that would otherwise remain undetected. The synergistic integration of these complementary modelling approaches resulted in significant enhancement of overall model performance and stability.

The CAMI-DM 2.0 model developed in this study ultimately incorporated 11 features identified through Strategy 1: age, sex, EF, SCR, HB, SHR, HR, SBP, Killip, MA, and PCI. Among these, classical features such as age, SCR, HR, SBP, and Killip overlapped with the prognostic components in the GRACE score, all of which had been well-established as significant prognostic factors for outcomes of AMI patients [22]. Age and sex, as fundamental demographic variables, are closely associated with adverse outcomes in AMI. Older patients often present with atypical symptoms and a higher burden of comorbidities, leading to increase in-hospital mortality [27,28]. Female patients also face a significantly higher risk of in-hospital death compared with their male counterparts [29,30]. The EF and Killip class reflect cardiac systolic and pump function; lower EF and higher Killip class typically indicate larger infarct size and more severe cardiac dysfunction, both strongly associated with increased mortality risk [31]. Elevated serum creatinine is indicative of renal impairment and has been widely validated as a high-risk factor for in-hospital mortality in AMI. In addition, patients with impaired renal function often have multivessel coronary artery disease and multiple chronic comorbidities, adversely affecting long-term prognosis [32,33]. Notably, SHR was consistently selected across all feature selection strategies, highlighting its unique predictive value in assessing in-hospital mortality risk among patients with diabetes. Although no universal consensus exists regarding the definition of stress hyperglycemia in AMI patients, SHR provides a standardized approach to quantify the deviation of glycemic status from baseline metabolism during acute stress, thereby offering superior risk stratification capability [34]. Our findings align with previous research: Xu et al. reported SHR as a significant predictor of in-hospital mortality in coronary artery disease patients, particularly those with DM or impaired glucose tolerance [35]. In a diabetic AMI population, Schmitz et al. showed that SHR was predictive of 28-day mortality, indicating its potential value in short-term risk stratification [36]. From a pathophysiological perspective, the metabolic stress state reflected by elevated SHR may influence clinical outcomes through multiple mechanisms. Hyperglycemia has been implicated in promoting atherosclerotic plaque instability and rupture, altering thrombus composition, and exacerbating thrombotic burden by disrupting the thrombogenic microenvironment [37–39]. It also impairs collateral circulation, contributing to inadequate perfusion of ischemic regions and increased infarct size [40,41]. Additionally, abnormally elevated glucose levels could activate the sympathetic nervous system, thereby contributing to adverse outcomes [42]. Furthermore, increased SHR reflects enhanced oxidative stress and inflammatory activity, exacerbating vascular endothelial dysfunction and prothrombotic states, ultimately resulting in reduced coronary perfusion, expanded infarct size, and worsened cardiac function [34]. To further evaluate the robustness of the models, exploratory subgroup analyses were conducted across different populations. The CAMI-DM models demonstrated relatively stable predictive performance across heterogeneous populations, including diabetic subgroups and enrolment phases. Minor differences in performance were observed within the same model across subgroups, suggesting that internal heterogeneity may contribute to minor variability in performance. Given the relatively small number of events in certain subgroups, these findings should be interpreted with caution.

This study carries certain implications for clinical practice, manifested in several key aspects. First, during the model development and preliminary validation phases, both CAMI-DM 1.0 and CAMI-DM 2.0 demonstrated superior predictive performance compared to the conventional GRACE score currently recommended in guidelines, potentially enabling clinicians to more accurately assess in-hospital mortality risk in AMI patients complicated by diabetes, thereby facilitating optimized individualized treatment decisions. Second, in terms of feature exploration, the application of machine learning techniques identified crucial predictive factors that were often not captured by traditional models—most notably the SHR—which might serve as potential therapeutic targets and provide new insights into the pathophysiological mechanisms underlying DM-complicated AMI. Third, through the SHAP analysis of CAMI-DM 2.0 and its TabNet sub-model, we were able to capture and visualize complex nonlinear relationships among features, offering clinicians more intuitive insights into the model’s decision-making process and increasing trust in the predictive outcomes. At the clinical application level, risk prediction models often face a fundamental trade-off between simplicity and predictive accuracy, making it challenging to achieve both simultaneously. With the introduction of high-performance algorithms such as deep learning, model structures have become increasingly complex, and the prediction process largely relies on electronic devices. As a result, it is difficult to perform “mental-calculation-style” rapid assessments at the bedside, which limits the applicability of such models in clinical scenarios that demand timeliness and operational simplicity. To address this, following the development of high-accuracy CAMI-DM 2.0, we created a more streamlined CAMI-DM 1.0 with simplified model structure and enhanced computational efficiency. Although CAMI-DM 1.0 sacrifices some predictive accuracy, it substantially enhances clinical accessibility and operational convenience. From another perspective, the additional predictive gains of CAMI-DM 2.0 come at the cost of reduced interpretability and increased implementation complexity, emphasizing the need to carefully balance accuracy and practicality. The two models are intended to accommodate different clinical scenarios, rather than treating CAMI-DM 2.0 as a straightforward upgrade of CAMI-DM 1.0. We envision that CAMI-DM 1.0 can be used for immediate risk stratification during emergency admission or bedside rapid assessment, allowing clinicians to make an initial judgment of the patient’s risk level. Subsequently, with the aid of in-hospital systems or automated tools, the CAMI-DM 2.0 can be applied to perform a more comprehensive and refined risk evaluation, thereby optimizing individualized decision-making and facilitating the effective integration of the model into actual clinical workflows.

When interpreting and applying the predictive models developed in this study, several limitations should be carefully considered: (1) Although the dataset used in this study encompassed multiple regions and three healthcare tiers across China, offering substantial representativeness, its generalizability to more primary care settings and to populations with diverse ethnic backgrounds may be limited. In addition, the clinical characteristics, management pathways, and treatment strategies for AMI patients with diabetes have continuously evolved over time; accordingly, the performance and validity of the models in a changing clinical landscape warrant ongoing assessment. While the models demonstrated stable performance in internal validation, their robustness and applicability still require further confirmation through external validation. (2) Due to temporal constraints in data collection and limited testing capabilities at some primary care institutions, several clinically significant biomarkers (such as B-type natriuretic peptide and high-sensitivity C-reactive protein) were not routinely available and thus could not be included in the modeling process. In addition, potentially clinically relevant variables, such as more detailed echocardiographic parameters, were not collected in the CAMI registry at the time. This omission may have impacted the model’s predictive performance to some degree. (3) While the SHAP method was employed to enhance model interpretability, the underlying prediction mechanisms of neural network-based submodules within our ensemble framework remain incompletely understood. Future model refinements or novel interpretability approaches may help address this limitation. (4) In CAMI-DM 2.0, the inclusion of treatment-related features is methodologically justified within its intended application scenario and assessment time point; nevertheless, the potential for causal confounding should still be carefully considered, as reperfusion therapy is critically important for patient outcomes, yet the factors underlying the decision to administer reperfusion are multifactorial. (5) The development of the models in this study primarily relied on structured tabular data, incorporating multidimensional features including medical history, physical examinations, laboratory parameters, and echocardiographic findings. However, imaging and other data modalities were not included. Future incorporation of multimodal heterogeneous information may further improve predictive accuracy and clinical generalizability.

## Conclusions

This study focused on the specific population of AMI patients with diabetes, and for the first time developed two dedicated models to predict in-hospital mortality risk. The CAMI-DM 1.0, a simplified risk score, requires only five key features to facilitate rapid risk assessment. The CAMI-DM 2.0, by contrast, is a fusion model based on 11 features and implemented via an ensemble of GLM and TabNet, delivering superior predictive performance alongside improved clinical interpretability enabled by SHAP. Both models outperformed the traditional GRACE score in terms of predictive accuracy. The tailored application of these models in different clinical settings may support more precise identification of high-risk diabetic AMI patients and facilitates personalized treatment strategies, potentially contributing to optimized clinical management.

## Supporting information

S1 File Study design and cohort derivation. (DOCX)

S2 File Feature selection strategy, composite glucose-related indices, definitions of candidate features. (DOCX)

S1 Fig The missing data across candidate features in the study population. (DOCX)

S2 Fig ROC curves of SHR, Glucose, and HbAle. (DOCX)

S3 Fig Feature Selection Strategy 1: Elastic Net. (DOCX)

S4 Fig Feature Selection Strategy 2: RFE. (DOCX)

S5 Fig Performance comparison of twelve machine learning models under different Feature Selection Strategy. (DOCX)

S6 Fig ROC curves of various model combinations based on the Stacking ensemble strategy. (DOCX)

S7 Fig Concordance between Sub-model Predictions and True Labels in the Training Set. (DOCX)

S8 Fig SHAP decision plot for CAMI-DM 2.0. (DOCX)

S9 Fig Performance of logistic regression models under different feature selection strategy in the training set. (DOCX)

S10 Fig Distribution of CAMI-DM 1.0 features in the training set. (DOCX)

S1 Table STROBE statement. (DOCX)

S2 Table List of candidate features. (DOCX)

S3 Table Baseline characteristics: Training vs test sets. (DOCX)

S4 Table Univariate logistic regression analysis. (DOCX)

S5 Table Derivation and scoring process of the CAMI-DM 1.0 Model. (DOCX)

S6 Table Exploratory subgroup analyses. (DOCX)

## Abbreviations

AMI: Acute myocardial infarction
AUROC: Area under the receiver operating characteristic curve
AUPRC: Area under the precision-recall curve
BMI: Body mass index
DCA: Decision curve analysis
DL: Deep learning
DM: Diabetes mellitus
DNN: Deep neural networks
EF: Ejection fraction
FP: False positives
FN: False negatives
GBM: Gradient Boosting Machine
GLM: Generalized Linear Model
HB: Hemoglobin
HbA1c: Glycated hemoglobin
HR: Heart rate
IDI: Integrated Discrimination Improvement
K ⁺: Serum potassium
KNN: K-Nearest Neighbors Classifier
LightGBM: Light Gradient Boosting Machine
MA: Malignant arrhythmia
ML: Machine learning
NSTEMI: Non–ST-segment elevation myocardial infarction
NPV: Negative predictive value
PCI: Percutaneous coronary intervention
PPV: Positive predictive value
RF: Random Forest
SBP: Systolic blood pressure
SCR: Serum creatinine
SHAP: Shapley Additive explanations
SHR: Stress hyperglycemia ratio
STEMI: ST-segment elevation myocardial infarction
SVM: Support Vector Machine
HF: Heart Failure
TN: True negatives
TP: True positives
TyG: Triglyceride-glucose index
