# Supplementary material for: CAMI-DM: Development and validation of a multi-algorithm model for in-hospital mortality risk prediction in diabetic patients with acute myocardial infarction — The China acute myocardial infarction registry
Source: PLOS Digit Health. 2026 Aug 7;5(8):e0001529. doi: 10.1371/journal.pdig.0001529 (PMC13450723; doi:10.1371/journal.pdig.0001529)
Supplement: Supplementary file 1 [file pdig.0001529.s001.docx]

**Study Design and Cohort Derivation**

**This study was conducted in two phases. Phase I spanned from January 2013 to September 2014 and enrolled all patients with acute myocardial infarction (AMI), including both ST-segment elevation myocardial infarction (STEMI) and non–ST-segment elevation myocardial infarction (NSTEMI), who were admitted within 7 days of symptom onset. Phase II extended from September 2014 to January 2016, during which enrollment was restricted to patients hospitalized within 3 days of symptom onset, including both STEMI and NSTEMI cases. According to the universally accepted definitions of AMI, a patient was confirmed to have AMI if they met at least one of the following criteria, which also served as the final inclusion criteria for this registry study: (1) Elevation of cardiac enzymes or biomarkers (preferably troponin), or a rise followed by a fall, with at least one measurement exceeding the 99th percentile of the upper reference limit, accompanied by at least one of the following indicators of myocardial ischemia: ① clinical symptoms of myocardial ischemia; ② new ischemic changes on electrocardiogram, including ST-T segment changes or new left bundle branch block; ③ pathological Q waves on electrocardiogram; ④ imaging evidence of new loss of myocardial viability or regional wall motion abnormality; ⑤ intracoronary thrombus demonstrated by coronary angiography or autopsy. (2) Sudden cardiac death, including cardiac arrest, often accompanied by symptoms suggestive of myocardial ischemia, possible new ST-segment elevation or left bundle branch block, and/or evidence of fresh coronary thrombus by angiography or autopsy, even if blood specimens could not be obtained or cardiac biomarkers were not yet detectable.**

**All confirmed AMI patients undergoing emergency or inpatient treatment were to be registered promptly. A unique patient ID was automatically generated upon submission of the baseline CRF, after which all clinical information during the treatment course was collected and entered item-by-item into the electronic CRF system. The key steps were as follows: (1) Initial CRF entry: Confirmed AMI patients received priority clinical care. Once stabilized, the responsible physician logged into the registry system or used offline software to complete the baseline CRF page and submit it online, which typically required 2–3 minutes. All eligible patients were to be reported within 24 hours of presentation or admission. (2) Ongoing data collection: Following baseline registration, all clinical information during the hospital stay—including treatment course, therapeutic outcomes, complications, and adverse events—was collected and entered electronically, either online or via offline software, and then uploaded. If certain information was unknown or uncertain, “unknown” was entered objectively. Complete data were expected upon discharge. (3) Exclusion after initial inclusion: If a patient initially diagnosed with AMI and entered into the registry was later determined not to have AMI, the patient status was marked as “excluded,” and the reason for exclusion was documented. (4) Discharge or death: After discharge or death, all CRF data were finalized and submitted. Quality control personnel at each site performed timely review, followed by secondary verification and data locking by the site principal investigator. (5) Follow-up: Survivors entered a follow-up program according to pre-specified protocols. Follow-up data were collected online, ensuring complete longitudinal information. The registry system included automated reminders for each scheduled follow-up visit. (6)Data management compliance: All data entry, submission, and management procedures were conducted in accordance with Good Clinical Practice (GCP) guidelines and the study-specific Standard Operating Procedures (SOPs).**

Patient discharge or death

Patients diagnosed with AMI

Itemized entry of electronic CRF and online submission

Informed consent signed; baseline page of electronic CRF submitted (within 24 hours)

Completion of all information collection during hospitalization

Review approved

Quality control staff / responsible personnel

Acute Myocardial Infarction Database (including baseline info at emergency visit and admission, complete hospitalization and discharge data, and follow-up information)

Emergency and inpatient treatment

Follow-up

Emergency care
