# Supplementary material for: CAMI-DM: Development and validation of a multi-algorithm model for in-hospital mortality risk prediction in diabetic patients with acute myocardial infarction — The China acute myocardial infarction registry
Source: PLOS Digit Health. 2026 Aug 7;5(8):e0001529. doi: 10.1371/journal.pdig.0001529 (PMC13450723; doi:10.1371/journal.pdig.0001529)
Supplement: Supplementary file 2 [file pdig.0001529.s002.docx]

**Feature Selection Strategy**

**All feature selection procedures were conducted strictly within the training set to avoid information leakage. Feature selection was not nested within cross-validation folds; instead, it was performed on the entire training set prior to subsequent model development and evaluation. A preliminary univariate analysis was conducted to exclude features not significantly associated with in-hospital mortality (p > 0.1). (1) Elastic Net Regularization: A series of 10-fold cross-validations was performed across a range of α values (representing different balances of L1 and L2 penalties). The combination of α and λ yielding the lowest mean cross-validation error was selected as the optimal hyperparameter set. Features with non-zero coefficients under the λ.1se criterion were retained from the entire training set for model development. (2) Recursive Feature Elimination (RFE): Feature importance was assessed using a random forest algorithm on the training set, followed by 5-fold cross-validation to iteratively eliminate the least important features. The number of features corresponding to the lowest cross-validation error was identified as optimal, and the top-ranked features based on mean decrease in Gini index were retained. (3) Consensus Feature Selection: To generate a simplified candidate feature set for model development while maintaining clinical interpretability, the intersection of features selected by Elastic Net and RFE was used. This approach reflects a semi-prespecified selection process, incorporating both algorithmic results and clinical considerations (excluding treatment-related features), including anticipated usage scenarios, rather than purely representing the “only optimal” or “most robust” set. This approach leans more toward a prespecified parameter-based selection process and involves a certain degree of subjective choice. It generates a parsimonious feature set by combining algorithmic results with consideration of clinical needs and intended usage scenarios. Furthermore, it should be noted that this cross-algorithm approach may exclude important features that are identified by only one method but are clinically relevant.**

**Composite Glucose-Related Indices**

**For the development of predictive models in AMI patients with diabetes, two composite glucose-related indices (SHR and TyG) were prioritized as candidate features based on several considerations. As described in the main text, prior studies have demonstrated that SHR and TyG, as composite markers of glucose metabolism, generally provide stronger predictive value for cardiovascular adverse outcomes and mortality than their individual components. In terms of biological plausibility, both indices more comprehensively capture stress hyperglycemia, insulin resistance, and broader metabolic dysregulation compared with single glucose measures or individual metabolic components, supporting their mechanistic relevance in AMI prognosis. In addition, these markers are readily available and computable within the CAMI dataset, making them suitable for inclusion as candidate features in predictive model development. Previous CAMI-based studies have reported the prognostic relevance of SHR or TyG in DM populations, demonstrating stronger predictive effects than single metabolic markers. in the present study, SHR and TyG were incorporated into a multivariable, multi-algorithm risk prediction framework specifically targeting AMI patients with diabetes, rather than solely validating single-marker associations.**

**Definitions of Candidate Features**

**Killip Classification**

**Killip classification refers to the grading of patients based on their clinical presentation upon arrival at the hospital, specifically for acute myocardial infarction. Class I: No pulmonary rales and no third heart sound; Class II: Pulmonary rales involving less than one-half of the lung fields, or the presence of a third heart sound; Class III: Pulmonary rales involving more than one-half of the lung fields; Class IV: Cardiogenic shock.**

**History of Hypertension**

**History of hypertension refers to hypertension occurring from birth to before the onset of acute myocardial infarction. Hypertension was defined as meeting any of the following criteria: (1) A previous diagnosis of hypertension, treated with medication, diet, and/or exercise; (2) Previous records showing systolic blood pressure >140 mmHg and/or diastolic blood pressure >90 mmHg in patients without diabetes or kidney disease; or at least two measurements of systolic blood pressure >130 mmHg and/or diastolic blood pressure >80 mmHg in patients with diabetes or kidney disease; (3) Current use of antihypertensive medications.**

**History of Dyslipidemia**

**History of dyslipidemia refers to dyslipidemia diagnosed by a physician and/or treatment initiated from birth to before the onset of acute myocardial infarction, including any of the following: (1) Total cholesterol >200 mg/dL (5.18 mmol/L); (2) Low-density lipoprotein cholesterol (LDL-C) ≥130 mg/dL (3.37 mmol/L); (3) High-density lipoprotein cholesterol (HDL-C) <40 mg/dL (1.04 mmol/L).**

**Family history of premature CAD**

**Family history refers to any first-degree relative, including parents, siblings, or children, with any of the following diagnoses before the age of 55 years in male relatives or before the age of 65 years in female relatives:** **(1) Angina pectoris; (2) Acute myocardial infarction; (3) Sudden cardiac death without an apparent cause; (4) Coronary artery bypass grafting; (5) Percutaneous coronary intervention.**

**Previous MI**

**History of prior myocardial infarction was defined as meeting any of the following criteria: (1) A previous diagnosis of acute myocardial infarction; (2) A history of elevated cardiac enzymes or troponin accompanied by symptoms of myocardial ischemia, electrocardiographic changes, or imaging abnormalities; (3) Abnormal pathological Q waves on electrocardiogram; (4)Imaging evidence from echocardiography, radionuclide myocardial imaging, CT, or MRI showing segmental wall motion abnormalities or irreversible perfusion defects, without other non-ischemic causes.**

**Previous HF**

**History of prior heart failure refers to heart failure occurring from birth to before the onset of acute myocardial infarction, with previous clinical symptoms including fatigue with mild activity, dyspnea frequently occurring in the supine position, or fluid retention; or physical examination findings including rales, jugular venous distension, pulmonary edema, or pulmonary edema on chest X-ray judged to be caused by cardiac dysfunction. A reduced ejection fraction without clinical evidence of heart failure was not considered heart failure.**

**Previous stroke**

**History of prior stroke refers to stroke occurring at any time from birth to before the onset of acute myocardial infarction. A history of stroke was defined as any definite neurological deficit caused by impaired cerebral blood supply that was not corrected within 24 hours.**

**Previous Peripheral Vascular Disease**

**History of peripheral vascular disease refers to peripheral arterial disease occurring from birth to before the onset of acute myocardial infarction, involving the upper or lower extremities, renal arteries, mesenteric arteries, or abdominal aortic system, including any of the following: (1) Intermittent claudication during exertion or at rest; (2) Amputation due to insufficient arterial blood supply; (3) Revascularization, bypass surgery, or interventional treatment of the extremities, excluding dialysis fistula procedures and vein stripping; (4) Documented treated or untreated aortic aneurysm; (5) Positive noninvasive test findings, such as ankle-brachial index ≤0.9; or ultrasound, magnetic resonance imaging, CT, or angiographic evidence of >50% diameter stenosis in any peripheral artery, such as renal, subclavian, femoral, or iliac arteries.**

**Previous Bleed**

**History of bleeding refers to a prior history of significant spontaneous bleeding occurring from birth to before the onset of acute myocardial infarction, requiring hospital evaluation and/or medication or specialized treatment. Surgery-related bleeding was not included.**

**Abnormal Q Waves**

**Abnormal Q waves refer to newly developed abnormal Q waves, defined as Q-wave duration ≥0.04 s and depth ≥ one-fourth of the following R wave, with notching or slurring of the Q wave, or a Q wave deeper than previously observed.**

**ST-Segment Elevation**

**ST-segment elevation refers to new ST-segment elevation at the J point in two contiguous leads, ≥0.1 mV; or in leads V2–V3, ≥0.2 mV in men aged ≥40 years, ≥0.25 mV in men aged <40 years, or ≥0.15 mV in women.**

**ST-Segment Depression**

**ST-segment depression refers to new horizontal or downsloping ST-segment depression ≥0.05 mV in two contiguous leads.**

**Emergency PCI**

**Emergency PCI treatment refers to emergency percutaneous coronary intervention performed for a completely occluded or near-occluded coronary artery in an emergency setting, including thrombus aspiration, balloon dilation, and stent implantation.**

**Emergency Reperfusion Therapy**

**Emergency reperfusion therapy includes thrombolysis and emergency coronary intervention, including percutaneous coronary intervention or coronary artery bypass grafting, aimed at reopening the acutely occluded coronary artery, restoring myocardial perfusion, salvaging ischemic myocardium, and reducing infarct size. It also improves hemodynamics, preserves cardiac function, and reduces the incidence of pump failure and in-hospital mortality.**
