# Supplementary material for: CAMI-DM: Development and validation of a multi-algorithm model for in-hospital mortality risk prediction in diabetic patients with acute myocardial infarction — The China acute myocardial infarction registry
Source: PLOS Digit Health. 2026 Aug 7;5(8):e0001529. doi: 10.1371/journal.pdig.0001529 (PMC13450723; doi:10.1371/journal.pdig.0001529)
Supplement: Supplementary file 3 [file pdig.0001529.s003.docx]

**Figure S1. The missing data across candidate features in the study population**

**#** **This figure illustrates the number of missing values for each of the 62 candidate features. The red vertical line marks the 20% missingness threshold. features with missingness above this threshold (n=9) were excluded.**

**#** **BMI, body mass index; SBP, systolic blood pressure; DBP, diastolic blood pressure; MAP, mean arterial pressure; HR, heart rate; K+, serum potassium; Na+, serum sodium; Cl-, serum chloride; WBC, white blood cell count; PLT, platelet count; HB, hemoglobin; SCR, serum creatinine; TC, total cholesterol; TG, triglyceride; HDL-C, high-density lipoprotein cholesterol; LDL-C, low-density lipoprotein cholesterol; Glu, admission blood glucose; HbA1c, glycated hemoglobin; TyG, triglyceride-glucose index; SHR, stress hyperglycemia ratio; beta, beta-blockers; RASS, renin–angiotensin system inhibitors; Statin, statins; P2Y12, P2Y12 inhibitors; Aspirin, aspirin use; EF, ejection fraction; LVEDd, left ventricular end-diastolic diameter; MA, malignant arrhythmia; MICA, myocardial infarction in the circumflex artery; STE, ST-segment elevation; STD, ST-segment depression; QW, pathological Q wave; AVB, atrioventricular block; NCLBBB, non-complete left bundle branch block; NCRBBB, non-complete right bundle branch block; Stroke, prior stroke; COPD, chronic obstructive pulmonary disease; HF, heart failure; PAD, peripheral artery disease; Cancerh, history of cancer; Bleedh, history of bleeding; DM, diabetes mellitus; DMT, diabetes treatment; ITDM, insulin-treated diabetes mellitus; HT, hypertension; HL, hyperlipidemia; Smoke, smoking history; FHCAD, family history of coronary artery disease; Anginah, history of angina; Mih, history of myocardial infarction; PCIh, prior percutaneous coronary intervention; CABGh, prior coronary artery bypass grafting; SyTD, symptom-to-door time.**
