# Supplementary material for: CAMI-DM: Development and validation of a multi-algorithm model for in-hospital mortality risk prediction in diabetic patients with acute myocardial infarction — The China acute myocardial infarction registry
Source: PLOS Digit Health. 2026 Aug 7;5(8):e0001529. doi: 10.1371/journal.pdig.0001529 (PMC13450723; doi:10.1371/journal.pdig.0001529)
Supplement: Supplementary file 4 [file pdig.0001529.s004.docx]

**Figure S2. ROC curves of SHR, Glucose, and HbA1c**

**# Event: In-hospital death; datasets: training set**

**# SHR, stress hyperglycemia ratio; Glu, admission blood glucose; HbA1c, glycated hemoglobin**
