# Supplementary material for: CAMI-DM: Development and validation of a multi-algorithm model for in-hospital mortality risk prediction in diabetic patients with acute myocardial infarction — The China acute myocardial infarction registry
Source: PLOS Digit Health. 2026 Aug 7;5(8):e0001529. doi: 10.1371/journal.pdig.0001529 (PMC13450723; doi:10.1371/journal.pdig.0001529)
Supplement: Supplementary file 5 [file pdig.0001529.s005.docx]

**Figure S3. Feature Selection Strategy 1: Elastic Net**

(1) **Cross-validation**
**and Coefficient path plot**

**#Left: Cross-validation curve of the Elastic Net model (α = 0.9), showing the binomial deviance across different values of the regularization parameter (λ).**

**#Right: Coefficient paths of selected variables as λ varies. As λ decreases, more variables enter the model, with greater shrinkage occurring at higher values of λ due to stronger penalization. The dashed lines indicate the optimal λ with minimum deviance (λₘᵢₙ, corresponding to 21 variables) and the λ within one standard error of the minimum (λ.₁ₛₑ, corresponding to 12 variables).**

**(2) Coefficients of features selected by** **Elastic Net (λ.1se)**

**# Positive coefficients (orange) indicate risk factors, while negative coefficients (blue) suggest protective factors. The magnitude of the coefficients reflects their relative contribution to the linear predictor of in-hospital mortality.**

**# EF, ejection fraction; HB, hemoglobin; HR, heart rate; MA, malignant arrhythmia; SBP, systolic blood pressure; SCR, serum creatinine; SHR, stress hyperglycemia ratio; PCI, percutaneous coronary intervention**
