# Supplementary material for: CAMI-DM: Development and validation of a multi-algorithm model for in-hospital mortality risk prediction in diabetic patients with acute myocardial infarction — The China acute myocardial infarction registry
Source: PLOS Digit Health. 2026 Aug 7;5(8):e0001529. doi: 10.1371/journal.pdig.0001529 (PMC13450723; doi:10.1371/journal.pdig.0001529)
Supplement: Supplementary file 6 [file pdig.0001529.s006.docx]

**Figure S4. Feature Selection Strategy 2: RFE**

**# Feature selection was conducted using recursive feature elimination (RFE) with 5-fold cross-validation, implemented via random forest algorithm. The left panel displays the cross-validation error curve, with the minimum error observed at 7 variables, indicating the optimal subset size. Subsequently, a full random forest model was refitted using all candidate variables to compute Gini-based importance scores. The top 7 variables ranked by mean decrease in Gini index are shown in the right panel.**

**# SCR, serum creatinine; SHR, stress hyperglycemia ratio; K+, serum potassium; EF, left ventricular ejection fraction; HR, heart rate; BMI, body mass index**
