# Supplementary material for: CAMI-DM: Development and validation of a multi-algorithm model for in-hospital mortality risk prediction in diabetic patients with acute myocardial infarction — The China acute myocardial infarction registry
Source: PLOS Digit Health. 2026 Aug 7;5(8):e0001529. doi: 10.1371/journal.pdig.0001529 (PMC13450723; doi:10.1371/journal.pdig.0001529)
Supplement: Supplementary file 7 [file pdig.0001529.s007.docx]

**Figure S5. Performance comparison of twelve machine learning models under different** **Feature Selection Strategy**

**(1)** **ROC curves of twelve machine learning models (Feature Selection Strategy2: RFE) in the test set**

**(2) ROC curves of twelve machine learning models (Feature Selection Strategy3: Consensus Feature) in the test set**
