# Supplementary material for: CAMI-DM: Development and validation of a multi-algorithm model for in-hospital mortality risk prediction in diabetic patients with acute myocardial infarction — The China acute myocardial infarction registry
Source: PLOS Digit Health. 2026 Aug 7;5(8):e0001529. doi: 10.1371/journal.pdig.0001529 (PMC13450723; doi:10.1371/journal.pdig.0001529)
Supplement: Supplementary file 8 [file pdig.0001529.s008.docx]

**Figure S6. ROC curves of various model combinations based on the Stacking ensemble strategy**

**# predicted probabilities of multiple base models were used as input features to train a logistic regression meta-learner, and ensemble performance was subsequently evaluated on the test set.**
