# Supplementary material for: CAMI-DM: Development and validation of a multi-algorithm model for in-hospital mortality risk prediction in diabetic patients with acute myocardial infarction — The China acute myocardial infarction registry
Source: PLOS Digit Health. 2026 Aug 7;5(8):e0001529. doi: 10.1371/journal.pdig.0001529 (PMC13450723; doi:10.1371/journal.pdig.0001529)
Supplement: Supplementary file 9 [file pdig.0001529.s009.docx]

**Figure S7. Concordance between Sub-model Predictions and True Labels in the Training Set**

**# The two sub-models used in CAMI-DM 2.0, namely the Generalized Linear Model (GLM) and TabNet.**

**#Left: Scatterplot showing the predicted probabilities from GLM (x-axis) and TabNet (y-axis), with color indicating the average probability of the two models.**

**#Right: The same scatterplot, colored by the true label (red = death, blue = survival), illustrating the spatial distribution of positive cases.**
