# Supplementary material for: CAMI-DM: Development and validation of a multi-algorithm model for in-hospital mortality risk prediction in diabetic patients with acute myocardial infarction — The China acute myocardial infarction registry
Source: PLOS Digit Health. 2026 Aug 7;5(8):e0001529. doi: 10.1371/journal.pdig.0001529 (PMC13450723; doi:10.1371/journal.pdig.0001529)
Supplement: Supplementary file 10 [file pdig.0001529.s010.docx]

**Figure S8. SHAP decision plot for CAMI-DM 2.0**

**# An exploration of the prediction pathway for the CAMI-DM 2.0 model was conducted using a SHAP decision plot, which visually illustrates how different feature combinations shape individual prediction outcomes. In the plot, each line represents a single patient, with blue lines indicating predicted survival and red lines indicating predicted mortality. The slope and inflection points of each line reflect both the direction and magnitude of each feature’s contribution, providing insight into the step-by-step reasoning behind the model’s prediction.**
