# Supplementary material for: CAMI-DM: Development and validation of a multi-algorithm model for in-hospital mortality risk prediction in diabetic patients with acute myocardial infarction — The China acute myocardial infarction registry
Source: PLOS Digit Health. 2026 Aug 7;5(8):e0001529. doi: 10.1371/journal.pdig.0001529 (PMC13450723; doi:10.1371/journal.pdig.0001529)
Supplement: Supplementary file 11 [file pdig.0001529.s011.docx]

**Figure S9. Performance of Logistic Regression Models under different Feature Selection Strategy in the Training Set**

**# Model 1, Model 2, and Model 3 represent logistic regression models constructed using feature sets from Feature Selection Strategy 1, 2, and 3, respectively. The GRACE score was included as a comparator.**

**# Model performance was evaluated on the training set using 5-fold cross-validation.**
