# Supplementary material for: CAMI-DM: Development and validation of a multi-algorithm model for in-hospital mortality risk prediction in diabetic patients with acute myocardial infarction — The China acute myocardial infarction registry
Source: PLOS Digit Health. 2026 Aug 7;5(8):e0001529. doi: 10.1371/journal.pdig.0001529 (PMC13450723; doi:10.1371/journal.pdig.0001529)
Supplement: Supplementary file 12 [file pdig.0001529.s012.docx]

**Figure S10. Distribution of CAMI-DM 1.0 Features in the Training Set**

**# This figure shows the distribution of the five variables used in the CAMI-DM 1.0 model—Age, EF (ejection fraction), SCR (serum creatinine), Glu (admission blood glucose), and HR (heart rate)—within the training dataset. The orange lines represent smoothed density curves, and the red dashed lines indicate the median values of each variable.**

**#** **As a simplified point-based scoring model designed for bedside use, CAMI-DM 1.0 converted continuous variables into categorical intervals to facilitate clinical calculation and memorability. The cut-points were determined with reference to the distribution, median value, and observed risk gradient of each feature in the training set, and were simplified while considering bedside operational convenience.**
