# Supplementary material for: CAMI-DM: Development and validation of a multi-algorithm model for in-hospital mortality risk prediction in diabetic patients with acute myocardial infarction — The China acute myocardial infarction registry
Source: PLOS Digit Health. 2026 Aug 7;5(8):e0001529. doi: 10.1371/journal.pdig.0001529 (PMC13450723; doi:10.1371/journal.pdig.0001529)
Supplement: Supplementary file 13 [file pdig.0001529.s013.docx]

**The reporting of this study followed the "Strengthening the Reporting of Observational Studies in Epidemiology" (STROBE) reporting guideline**

| Table S1. STROBE Statement—checklist of items that should be included in reports of observational studies | | | |
| --- | --- | --- | --- |
|  | **Item** | **Recommendation** | **Page** |
| Title and abstract | 1 | (a) Indicate the study’s design with a commonly used term in the title or the abstract | 1,2 |
|  |  | (b) Provide in the abstract an informative and balanced summary of what was done and what was found | 1,2 |
| Introduction |  |  |  |
| Background/rationale | 2 | Explain the scientific background and rationale for the investigation being reported | 4-6 |
| Objectives | 3 | State specific objectives, including any prespecified hypotheses | 6 |
| Methods |  |  |  |
| Study design | 4 | Present key elements of study design early in the paper | 6-7 |
| Setting | 5 | Describe the setting, locations, and relevant dates, including periods of recruitment, exposure, follow-up, and data collection | 6-7 |
| Participants | 6 | (a) Cohort study—Give the eligibility criteria, and the sources and methods of selection of participants. Describe methods of follow-up | 6-7 |
|  |  | (b) Cohort study—For matched studies, give matching criteria and number of exposed and unexposed | NA |
| Variables | 7 | Clearly define all outcomes, exposures, predictors, potential confounders, and effect modifiers. Give diagnostic criteria, if applicable | 6-10 |
| Data sources/ measurement | 8 | For each variable of interest, give sources of data and details of methods of assessment (measurement). Describe comparability of assessment methods if there is more than one group. | 6-7 |
| Bias | 9 | Describe any efforts to address potential sources of bias | 11 |
| Study size | 10 | Explain how the study size was arrived at | 6-11 |
| Quantitative variables | 11 | Explain how quantitative variables were handled in the analyses. If applicable, describe which groupings were chosen and why | 6-11 |
| Statistical methods | 12 | (a) Describe all statistical methods, including those used to control for confounding | 6-11 |
|  |  | (b) Describe any methods used to examine subgroups and interactions | 6-11 |
|  |  | (c) Explain how missing data were addressed | 6-11 |
|  |  | (d) Cohort study—If applicable, explain how loss to follow-up was addressed | 6-11 |
|  |  | (e) Describe any sensitivity analyses | 11 |
| Results |  |  |  |
| Participants | 13 | (a) Report numbers of individuals at each stage of study—eg numbers potentially eligible, examined for eligibility, confirmed eligible, included in the study, completing follow-up, and analyzed | 12 |
|  |  | (b) Give reasons for non-participation at each stage |  |
|  |  | (c) Consider use of a flow diagram |  |
| Descriptive data | 14 | (a) Give characteristics of study participants (eg. Demographic, clinical, social) and information on exposures and potential confounders | Table 1, Table S3 |
|  |  | (b) Indicate number of participants with missing data for each variable of interest | 12 |
|  |  | (c) Cohort study—Summarize follow-up time (eg, average and total amount) | 12 |
| Outcome data | 15 | Cohort study—Report numbers of outcome events or summary measures over time | 12 |
| Main results | 16 | (a) Give unadjusted estimates and, if applicable, confounder- adjusted estimates and their precision (eg, 95% confidence interval). Make clear which confounders were adjusted for and why they were included | 12-17 |
|  |  | (b) Report category boundaries when continuous variables were categorized | 12-17 |
|  |  | (c) If relevant, consider translating estimates of relative risk into absolute risk for a meaningful time period | NA |
| Other analyses | 17 | Report other analyses done—eg analyses of subgroups and interactions, and sensitivity analyses | NA |
| Discussion |  |  |  |
| Key results | 18 | Summarize key results with reference to study objectives | 18-20 |
| Limitations | 19 | Discuss limitations of the study, taking into account sources of potential bias or imprecision. Discuss both direction and magnitude of any potential bias | 21 |
| Interpretation | 20 | Give a cautious overall interpretation of results considering objectives, limitations, multiplicity of analyses, results from similar studies, and other relevant evidence | 21 |
| Generalizability | 21 | Discuss the generalizability (external validity) of the study results | 21 |
| Other information |  |  |  |
| Funding | 22 | Give the source of funding and the role of the funders for the present study and, if applicable, for the original study on which the present article is based | 1,24 |
