# Supplementary material for: CAMI-DM: Development and validation of a multi-algorithm model for in-hospital mortality risk prediction in diabetic patients with acute myocardial infarction — The China acute myocardial infarction registry
Source: PLOS Digit Health. 2026 Aug 7;5(8):e0001529. doi: 10.1371/journal.pdig.0001529 (PMC13450723; doi:10.1371/journal.pdig.0001529)
Supplement: Supplementary file 14 [file pdig.0001529.s014.docx]

| **List of 62 Candidate features Considered for Modeling** | |
| --- | --- |
| **Demographics** | Age, Sex, BMI, Smoke |
| **Physical Examination** | HR, SBP, SBP Range (≤90 / 90–180 / ≥180 mmHg), DBP, MAP, Killip |
| **Medical History** | Hypertension, Dyslipidemia, Previous angina, Previous MI, Previous HF, Family history of premature CAD, Previous PCI, Previous CABG, Previous stroke, Previous COPD, Previous PAD, Previous digestive ulcer, Previous Bleed, Previous Cancer |
| **Echocardiographic Parameters** | LVEDd, EF |
| **Laboratory Data** | SCR, HB, PLT, WBC, K⁺, Na⁺, Cl⁻, TC, TG, LDL-C, HDL-C |
| **ECG Findings** | NCLBBB, NCRBBB, ST-segment elevation, ST-segment depression, Pathological Q wave, AVB |
| **Diabetes-Related** **variables** | Duration of DM, DM medications (Insulin), Glu, HbA1C |
| **Composite Indices** | TyG, SHR |
| **MI-Related Variables** | MI Type (NSTEMI/STEMI), Time to hospital (>6 hours), MI with cardiac arrest, Malignant arrhythmia |
| **Medications** | Aspirin, P2Y12 inhibitor, beta-blocker, RAS inhibitors, Statin |
| **Emergency Reperfusion Therapy** | Emergency Reperfusion Therapy, Thrombolytic Therapy, PCI, CABG |

**Table S2. List of Candidate features**

**#Abbreviations: BMI = Body Mass Index; HR = Heart Rate; SBP = Systolic Blood Pressure; DBP = Diastolic Blood Pressure; MAP = Mean Arterial Pressure; LVEDd = Left Ventricular End-Diastolic Diameter; EF = Ejection Fraction; SCR = Serum Creatinine; HB = Hemoglobin; PLT = Platelet Count; WBC = White Blood Cell Count; TC = Total Cholesterol; TG = Triglycerides; LDL-C = Low-Density Lipoprotein Cholesterol; HDL-C = High-Density Lipoprotein Cholesterol; TyG = Triglyceride-Glucose Index; SHR = Stress Hyperglycemia Ratio; AVB = Atrioventricular Block; PCI = Percutaneous Coronary Intervention; CABG = Coronary Artery Bypass Grafting; RAS = Renin-Angiotensin System.**
