# Supplementary material for: CAMI-DM: Development and validation of a multi-algorithm model for in-hospital mortality risk prediction in diabetic patients with acute myocardial infarction — The China acute myocardial infarction registry
Source: PLOS Digit Health. 2026 Aug 7;5(8):e0001529. doi: 10.1371/journal.pdig.0001529 (PMC13450723; doi:10.1371/journal.pdig.0001529)
Supplement: Supplementary file 15 [file pdig.0001529.s015.docx]

**Table S3. Baseline Characteristics: Training vs Test Sets**

| **Characteristic** | | **Overall**  N = 6,091 | **Training-set**  N = 4,872 | **Test-set**  N = 1,219 | **p-value** |
| --- | --- | --- | --- | --- | --- |
| **Demographics** | |  |  |  |  |
| **Age (years), Mean (SD)** | | 63 (12) | 63 (12) | 63 (12) | 0.3^1^ |
| **Sex, Male, n (%)** | | 4,262 (70.0%) | 3,398 (69.7%) | 864 (70.9%) | 0.4^2^ |
| **BMI (kg/mp2), Median (IQR)** | | 24.5 (22.6, 26.6) | 24.5 (22.7, 26.6) | 24.4 (22.5, 26.3) | 0.046^1^ |
| **Smoking status, Current, n (%)** | | 2,370 (38.9%) | 1,882 (38.6%) | 488 (40.0%) | 0.4^2^ |
| **Medical history** | |  |  |  |  |
| **Hypertension, n (%)** | | 3,783 (62.1%) | 3,011 (61.8%) | 772 (63.3%) | 0.3^2^ |
| **Dyslipidemia, n (%)** | | 4,941 (81.1%) | 3,942 (80.9%) | 999 (82.0%) | 0.4^2^ |
| **Previous angina, n (%)** | | 1,724 (35.7%) | 1,379 (35.8%) | 345 (35.6%) | >0.9^2^ |
| **Previous MI (>1 month), n (%)** | | 566 (11.8%) | 458 (12.0%) | 108 (11.3%) | 0.5^2^ |
| **Previous HF, n (%)** | | 212 (3.5%) | 170 (3.5%) | 42 (3.4%) | >0.9^2^ |
| **Family history of premature CAD, n (%)** | | 297 (4.9%) | 241 (4.9%) | 56 (4.6%) | 0.6^2^ |
| **Previous PCI, n (%)** | | 465 (7.6%) | 375 (7.7%) | 90 (7.4%) | 0.7^2^ |
| **Previous CABG, n (%)** | | 55 (0.9%) | 43 (0.9%) | 12 (1.0%) | 0.7^2^ |
| **Previous stroke, n (%)** | | 660 (10.8%) | 522 (10.7%) | 138 (11.3%) | 0.5^2^ |
| **Previous COPD, n (%)** | | 111 (1.8%) | 84 (1.7%) | 27 (2.2%) | 0.3^2^ |
| **Previous PAD, n (%)** | | 82 (1.3%) | 65 (1.3%) | 17 (1.4%) | 0.9^2^ |
| **Previous digestive ulcer, n (%)** | | 118 (3.2%) | 87 (3.0%) | 31 (4.2%) | 0.10^2^ |
| **Previous Bleed, n (%)** | | 62 (1.7%) | 46 (1.6%) | 16 (2.2%) | 0.3^2^ |
| **Previous Cancer, n (%)** | | 100 (1.6%) | 73 (1.5%) | 27 (2.2%) | 0.078^2^ |
| **Echocardiographic parameters** | |  |  |  |  |
| **LVEDd (mm), Median (IQR)** | | 50 (46, 54) | 50 (46, 54) | 50.0 (46, 54) | 0.3^1^ |
| **EF (%), Mean (SD)** | | 53 (10) | 53 (10) | 53 (11) | >0.9^1^ |
| **Laboratory data** | |  |  |  |  |
| **SCR (μmol/L), Median (IQR)** | | 76 (62, 94) | 76 (63, 93) | 76 (61, 95) | >0.9^1^ |
| **HB (g/L), Mean (SD)** | | 135 (20) | 135 (20) | 134 (21) | >0.9^1^ |
| **PLT (10^9/L), Median (IQR)** | | 205 (168, 248) | 204 (167, 247) | 208 (171, 250) | 0.2^1^ |
| **WBC (10^9/L), Median (IQR)** | | 9.6 (7.6, 12.0) | 9.6 (7.6, 12.0) | 9.6 (7.6, 12.0) | 0.8^1^ |
| **K+ (mmol/L), Mean (SD)** | | 4.04 (0.51) | 4.04 (0.51) | 4.03 (0.52) | 0.6^1^ |
| **Na+ (mmol/L), Mean (SD)** | | 138.3 (4.2) | 138.3 (4.2) | 138.4 (4.3) | 0.5^1^ |
| **Cl- (mmol/L), Mean (SD)** | | 101.9 (4.8) | 101.9 (4.8) | 101.9 (4.4) | 0.5^1^ |
| **TC (mmol/L), Median (IQR)** | | 4.50 (3.77, 5.30) | 4.50 (3.78, 5.30) | 4.49 (3.74, 5.34) | >0.9^1^ |
| **TG (mmol/L), Median (IQR)** | | 1.57 (1.11, 2.30) | 1.56 (1.11, 2.31) | 1.58 (1.12, 2.29) | 0.8^1^ |
| **LDL-C (mmol/L), Median (IQR)** | | 2.70 (2.14, 3.37) | 2.71 (2.14, 3.37) | 2.66 (2.13, 3.37) | 0.3^1^ |
| **HDL-C (mmol/L), Median (IQR)** | | 1.00 (0.80, 1.20) | 1.00 (0.80, 1.20) | 1.00 (0.80, 1.20) | 0.3^1^ |
| **Diabetes-Related variables** | |  |  |  |  |
| **Duration of DM, Median (IQR)** | | 2 (0, 9) | 2 (0, 9) | 2 (0, 8) | 0.6^1^ |
| **DM medications (Insulin), n (%)** | | 1,169.0 (19.2%) | 929.0 (19.1%) | 240.0 (19.7%) | 0.6^2^ |
| **Glu (mmol/L), Median (IQR)** | | 9.8 (7.3, 13.3) | 9.8 (7.4, 13.4) | 9.7 (7.3, 13.1) | 0.3^1^ |
| **HbA1C (%), Median (IQR)** | | 7.5 (6.7, 9.0) | 7.5 (6.7, 9.0) | 7.6 (6.8, 9.0) | 0.8^1^ |
| **Composite indices** | |  |  |  |  |
| **SHR, Median (IQR)** | | 1.00 (0.81, 1.27) | 1.00 (0.81, 1.28) | 0.98 (0.79, 1.25) | 0.077^1^ |
| **TyG, Median (IQR)** | | 9.44 (8.97, 9.95) | 9.44 (8.97, 9.96) | 9.46 (8.98, 9.91) | 0.7^1^ |
| **Physical Examination** | |  |  |  |  |
| **HR (beats/min), Mean (SD)** | | 80 (18) | 80 (18) | 80 (19) | 0.9^1^ |
| **SBP (mmHg), Mean (SD)** | | 131 (26) | 132 (26) | 131 (25) | 0.6^1^ |
| **SBP Range, n (%)** | |  |  |  | 0.7^2^ |
| 90-180mmHg | | 5,574 (91.5%) | 4,463 (91.6%) | 1,111 (91.1%) |  |
| ≥180mmHg | | 267 (4.4%) | 214 (4.4%) | 53 (4.3%) |  |
| ≤90mmHg | | 250 (4.1%) | 195 (4.0%) | 55 (4.5%) |  |
| **DBP (mmHg), Mean (SD)** | | 79 (15) | 79 (15) | 79 (15) | 0.8^1^ |
| **MAP (mmHg), Mean (SD)** | | 97 (17) | 97 (17) | 96 (17) | 0.7^1^ |
| **Killip classification, n (%)** | |  |  |  | 0.3^2^ |
| I | | 4,417 (72.5%) | 3,523 (72.3%) | 894 (73.3%) |  |
| II | | 1,042 (17.1%) | 852 (17.5%) | 190 (15.6%) |  |
| III | | 344 (5.6%) | 267 (5.5%) | 77 (6.3%) |  |
| IV | | 288 (4.7%) | 230 (4.7%) | 58 (4.8%) |  |
| **MI-related variables** | |  |  |  |  |
| **MI Type, n (%)** | |  |  |  | 0.9^2^ |
| NSTEMI | | 1,758 (28.9%) | 1,404 (28.8%) | 354 (29.0%) |  |
| STEMI | | 4,333 (71.1%) | 3,468 (71.2%) | 865 (71.0%) |  |
| **Time to hospital (>6 hours), n (%)** | | 3,389 (55.6%) | 2,706 (55.5%) | 683 (56.0%) | 0.82 |
| **MI with cardiac arrest, n (%)** | | 53 (0.9%) | 36 (0.7%) | 17 (1.4%) | 0.027^2^ |
| **Malignant arrhythmia, n (%)** | | 237 (3.9%) | 180 (3.7%) | 57 (4.7%) | 0.11^2^ |
| **Electrocardiographic findings** | |  |  |  |  |
| **NCLBBB, n (%)** | | 59 (1.0%) | 47 (1.0%) | 12 (1.0%) | >0.9^2^ |
| **NCRBBB, n (%)** | | 136 (2.2%) | 110 (2.3%) | 26 (2.1%) | 0.8^2^ |
| **ST-segment elevation, n (%)** | | 3,964 (65.1%) | 3,183 (65.3%) | 781 (64.1%) | 0.4^2^ |
| **ST-segment depression, n (%)** | | 1,203 (19.8%) | 957 (19.6%) | 246 (20.2%) | 0.7^2^ |
| **Pathological Q wave, n (%)** | | 1,118 (18.4%) | 891 (18.3%) | 227 (18.6%) | 0.8^2^ |
| **AVB, n (%)** | | 106 (1.7%) | 90 (1.8%) | 16 (1.3%) | 0.2^2^ |
| **Medications** | |  |  |  |  |
| **Aspirin, n (%)** | | 5,878 (96.5%) | 4,706 (96.6%) | 1,172 (96.1%) | 0.4^2^ |
| **P2Y₁₂ inhibitor, n (%)** | | 5,953 (97.7%) | 4,755 (97.6%) | 1,198 (98.3%) | 0.2^2^ |
| **β-blocker, n (%)** | | 4,261 (70.0%) | 3,406 (69.9%) | 855 (70.1%) | 0.9^2^ |
| **RAS inhibitor, n (%)** | | 4,415 (72.5%) | 3,528 (72.4%) | 887 (72.8%) | 0.8^2^ |
| **Statin, n (%)** | | 5,915 (97.1%) | 4,721 (96.9%) | 1,194 (97.9%) | 0.051^2^ |
| **Emergency Reperfusion Therapy, n (%)** | | 2,736 (44.9%) | 2,201 (45.2%) | 535 (43.9%) | 0.4^2^ |
| **Thrombolytic Therapy, n (%)** | | 479 (7.9%) | 397 (8.1%) | 82 (6.7%) | 0.10^2^ |
| **PCI, n (%)** | | 2,330 (38.3%) | 1,870 (38.4%) | 460 (37.7%) | 0.7^2^ |
| **CABG, n (%)** | | 6 (0.1%) | 2 (0.0%) | 4 (0.3%) | 0.017^3^ |
| **Death, n (%)** | | 293 (4.8%) | 228 (4.7%) | 65 (5.3%) | 0.3^2^ |
| ^1^Wilcoxon rank sum test |  |  |  |  |  |
| ^2^Pearson's Chi-squared test |  |  |  |  |  |
| ^3^Fisher's exact test |  |  |  |  |  |
