# Supplementary material for: CAMI-DM: Development and validation of a multi-algorithm model for in-hospital mortality risk prediction in diabetic patients with acute myocardial infarction — The China acute myocardial infarction registry
Source: PLOS Digit Health. 2026 Aug 7;5(8):e0001529. doi: 10.1371/journal.pdig.0001529 (PMC13450723; doi:10.1371/journal.pdig.0001529)
Supplement: Supplementary file 16 [file pdig.0001529.s016.docx]

**Table S4. Univariate logistic regression analysis**

|  | **Characteristic** | **OR** | **OR 95%CI** | **p-value** |
| --- | --- | --- | --- | --- |
|  | **Demographics** |  |  |  |
|  | **Age** | **1.066** | **(1.054-1.078)** | **<0.001** |
|  | **Sex (male vs female)** | **0.383** | **(0.302-0.485)** | **<0.001** |
|  | **BMI** | **0.909** | **(0.874-0.946)** | **<0.001** |
|  | **Smoke (Current vs Previous/Never)** | **0.423** | **(0.319-0.561)** | **<0.001** |
|  | **Medical history** |  |  |  |
|  | **Hypertension** | **1.352** | **(1.050-1.740)** | **0.019** |
|  | **Dyslipidemia** | **0.637** | **(0.487-0.833)** | **<0.001** |
|  | **Previous HF** | **2.938** | **(1.914-4.509)** | **<0.001** |
|  | **Family history of premature CAD** | **0.679** | **(0.357-1.289)** | **0.236** |
|  | **Previous PCI** | **0.459** | **(0.249-0.845)** | **0.012** |
|  | **Previous CABG** | **0.364** | **(0.050-2.642)** | **0.318** |
|  | **Previous stroke** | **2.231** | **(1.659-3.000)** | **<0.001** |
|  | **Previous COPD** | **1.340** | **(0.618-2.907)** | **0.459** |
|  | **Previous PAD** | **1.290** | **(0.518-3.212)** | **0.584** |
|  | **Previous Cancer** | **1.501** | **(0.690-3.267)** | **0.305** |
| **Echocardiographic parameters** | |  |  |  |
| **LVEDd** | | **1.022** | **(1.005-1.040)** | **0.013** |
| **EF** | | **0.938** | **(0.927-0.948)** | **<0.001** |
| **Laboratory data** | |  |  |  |
| **SCR (μmol/L)** | | **1.008** | **(1.006-1.009)** | **<0.001** |
| **HB (g/L)** | | **0.976** | **(0.970-0.981)** | **<0.001** |
| **K+ (mmol/L)** | | **1.597** | **(1.290-1.977)** | **<0.001** |
| **TG (mmol/L)** | | **0.850** | **(0.765-0.946)** | **0.003** |
| **LDL-C (mmol/L)** | | **0.989** | **(0.876-1.117)** | **0.863** |
| **HDL-C (mmol/L)** | | **1.164** | **(0.884-1.533)** | **0.281** |
|  | **Diabetes-Related variables** |  |  |  |
|  | **Duration of DM** | **1.038** | **(1.023-1.054)** | **<0.001** |
|  | **DM medications (Insulin vs. non-Insulin)** | **1.162** | **(0.873-1.547)** | **0.304** |
|  | **Glu (mmol/L)** | **1.092** | **(1.072-1.113)** | **<0.001** |
|  | **HbA1C (%)** | **1.065** | **(1.002-1.132)** | **0.043** |
|  | **Composite indices** |  |  |  |
|  | **SHR** | **2.603** | **(2.113-3.206)** | **<0.001** |
|  | **TyG** | **1.114** | **(0.956-1.299)** | **0.166** |
|  | **Physical Examination** |  |  |  |
|  | **HR (beats/min)** | **1.024** | **(1.019-1.030)** | **<0.001** |
|  | **SBP (mmHg)** | **0.984** | **(0.979-0.989)** | **<0.001** |
|  | **SBP Range, (≥180mmHg vs. 90-180mmHg)** | **0.576** | **(0.269-1.233)** | **0.155** |
|  | **SBP Range, (≤90mmHg vs. 90-180mmHg)** | **3.715** | **(2.562-5.386)** | **<0.001** |
|  | **DBP (mmHg)** | **0.976** | **(0.968-0.984)** | **<0.001** |
|  | **MAP (mmHg)** | **0.966** | **(0.954-0.981)** | **<0.001** |
|  | **Killip (II vs. I)** | **2.228** | **(1.632-3.041)** | **<0.001** |
|  | **Killip (III vs. I)** | **4.555** | **(3.131-6.627)** | **<0.001** |
|  | **Killip (IV vs. I)** | **10.293** | **(7.417-14.284)** | **<0.001** |
|  | **MI-related variables** |  |  |  |
|  | **MI Type, (STEMI vs. NSTEMI)** | **0.942** | **(0.729-1.218)** | **0.650** |
|  | **Time to hospital, (≥6h vs. 0-6h)** | **1.475** | **(1.155-1.885)** | **0.002** |
|  | **MI with cardiac arrest** | **4.729** | **(2.352-9.508)** | **<0.001** |
|  | **Malignant arrhythmia** | **3.758** | **(2.570-5.495)** | **<0.001** |
|  | **Electrocardiographic findings** |  |  |  |
|  | **NCLBBB** | **3.163** | **(1.487-6.728)** | **0.003** |
|  | **NCRBBB** | **2.335** | **(1.326-4.112)** | **0.003** |
|  | **STE** | **1.037** | **(0.810-1.329)** | **0.771** |
|  | **STD** | **1.535** | **(1.176-2.003)** | **0.002** |
|  | **QW** | **1.128** | **(0.842-1.513)** | **0.420** |
|  | **AVB** | **3.951** | **(2.319-6.730)** | **<0.001** |
|  | **Medication** |  |  |  |
|  | **Aspirin** | **0.327** | **(0.214-0.498)** | **<0.001** |
|  | **P2Y₁₂ inhibitor** | **0.323** | **(0.194-0.538)** | **<0.001** |
|  | **β-blocker** | **0.389** | **(0.307-0.492)** | **<0.001** |
|  | **RAS inhibitor** | **0.351** | **(0.277-0.444)** | **<0.001** |
|  | **Statin** | **0.226** | **(0.150-0.342)** | **<0.001** |
|  | **Emergency Reperfusion Therapy** |  |  |  |
|  | **Thrombolytic Therapy** | **0.376** | **(0.286-0.494)** | **<0.001** |
|  | **PCI** | **0.575** | **(0.334-0.993)** | **0.047** |
|  | **CABG** | **0.383** | **(0.286-0.513)** | **<0.001** |

**#Event: In-hospital death;**
