# Supplementary material for: CAMI-DM: Development and validation of a multi-algorithm model for in-hospital mortality risk prediction in diabetic patients with acute myocardial infarction — The China acute myocardial infarction registry
Source: PLOS Digit Health. 2026 Aug 7;5(8):e0001529. doi: 10.1371/journal.pdig.0001529 (PMC13450723; doi:10.1371/journal.pdig.0001529)
Supplement: Supplementary file 17 [file pdig.0001529.s017.docx]

**Table S5. Derivation and Scoring Process of the CAMI-DM 1.0 Model**

| **Variable** | **Range_or_Unit** | **Representative_Value** | **Delta_From_Baseline** | **Beta** | **Beta_x_Delta** | **Raw_Score adjust** | **Final_Score（*2）** |
| --- | --- | --- | --- | --- | --- | --- | --- |
| **Age** | **0-50** | **45** | **0** | **0.0542** | **0** | **0** | **0** |
|  | **50-60** | **55** | **10** | **0.0542** | **0.542** | **0.5** | **1** |
|  | **60-70** | **65** | **20** | **0.0542** | **1.084** | **1** | **2** |
|  | **70-80** | **75** | **30** | **0.0542** | **1.626** | **1.5** | **3** |
|  | **>80** | **85** | **40** | **0.0542** | **2.168** | **2** | **4** |
| **EF** | **>50%** | **60** | **0** | **-0.0453** | **0** | **0** | **0** |
|  | **40-50%** | **45** | **-15** | **-0.0453** | **0.6795** | **0.5** | **1** |
|  | **30-40%** | **35** | **-25** | **-0.0453** | **1.1325** | **1** | **2** |
|  | **20-30%** | **25** | **-35** | **-0.0453** | **1.5855** | **1.5** | **3** |
|  | **＜20%** | **10** | **-50** | **-0.0453** | **2.265** | **2** | **4** |
| **HR** | **0-50** | **25** | **0** | **0.00798** | **0** | **0** | **0** |
|  | **50-100** | **75** | **50** | **0.00798** | **0.399** | **0.5** | **1** |
|  | **100-150** | **125** | **100** | **0.00798** | **0.798** | **1** | **2** |
|  | **150-200** | **175** | **150** | **0.00798** | **1.197** | **1.5** | **3** |
|  | **>200** | **250** | **225** | **0.00798** | **1.7955** | **2** | **4** |
| **Glu** | **0-10** | **5** | **0** | **0.0818** | **0** | **0** | **0** |
|  | **10-15.** | **12.5** | **7.5** | **0.0818** | **0.6135** | **0.5** | **1** |
|  | **15-20** | **17.5** | **12.5** | **0.0818** | **1.0225** | **1** | **2** |
|  | **20-25** | **22.5** | **17.5** | **0.0818** | **1.4315** | **1.5** | **3** |
|  | **＞25** | **30** | **25** | **0.0818** | **2.045** | **2** | **4** |
| **SCR** | **0-100** | **50** | **0** | **0.00529** | **0** | **0** | **0** |
|  | **100-200** | **150** | **100** | **0.00529** | **0.529** | **0.5** | **1** |
|  | **>200** | **400** | **350** | **0.00529** | **1.8515** | **2** | **4** |

**# This table illustrates the development of the CAMI-DM 1.0 point-based scoring model based on five key variables: Age, EF (ejection fraction), HR (heart rate), Glu (admission blood glucose), and SCR (serum creatinine). For each variable, clinically meaningful intervals were defined, and a representative value was assigned. The difference between the selected value and the reference value was multiplied by the corresponding β coefficient from the logistic regression model. The resulting product was then rounded to the nearest half-integer and multiplied by 2 to obtain the final score, facilitating easier clinical interpretation and use.**
