# Supplementary material for: CAMI-DM: Development and validation of a multi-algorithm model for in-hospital mortality risk prediction in diabetic patients with acute myocardial infarction — The China acute myocardial infarction registry
Source: PLOS Digit Health. 2026 Aug 7;5(8):e0001529. doi: 10.1371/journal.pdig.0001529 (PMC13450723; doi:10.1371/journal.pdig.0001529)
Supplement: Supplementary file 18 [file pdig.0001529.s018.docx]

**Table S6. Exploratory subgroup analyses**

| **Subgroup** | **Events / Total (n, %)** | **Model** | **AUROC (95% CI)** | **AUPRC (95% CI)** | **Accuracy** | **Sensitivity** | **Specificity** | **PPV** | **NPV** |
| --- | --- | --- | --- | --- | --- | --- | --- | --- | --- |
| Newly recognized diabetes | 24/456 (5.26%) | CAMI-DM 2.0 | 0.873 (0.797–0.936) | 0.409 (0.262–0.611) | 0.818 | 0.792 | 0.819 | 0.195 | 0.979 |
|  |  | CAMI-DM 1.0 | 0.803 (0.714–0.881) | 0.278 (0.151–0.445) | 0.811 | 0.708 | 0.817 | 0.177 | 0.972 |
|  |  | GRACE Score | 0.755 (0.650–0.848) | 0.253 (0.140–0.430) | 0.719 | 0.708 | 0.720 | 0.123 | 0.966 |
| Previous diabetes | 41/763 (5.37%) | CAMI-DM 2.0 | 0.876 (0.840–0.911) | 0.371 (0.260–0.495) | 0.761 | 0.854 | 0.756 | 0.166 | 0.983 |
|  |  | CAMI-DM 1.0 | 0.832 (0.782–0.879) | 0.298 (0.196–0.427) | 0.730 | 0.727 | 0.747 | 0.140 | 0.974 |
|  |  | GRACE Score | 0.767 (0.690–0.834) | 0.179 (0.121–0.268) | 0.742 | 0.741 | 0.741 | 0.142 | 0.972 |
| Phase I | 18/436 (4.13%) | CAMI-DM 2.0 | 0.855 (0.773–0.927) | 0.470 (0.282–0.652) | 0.734 | 0.833 | 0.730 | 0.117 | 0.982 |
|  |  | CAMI-DM 1.0 | 0.823 (0.724–0.910) | 0.441 (0.258–0.648) | 0.780 | 0.667 | 0.785 | 0.118 | 0.976 |
|  |  | GRACE Score | 0.671 (0.531–0.799) | 0.138 (0.071–0.288) | 0.700 | 0.611 | 0.703 | 0.081 | 0.967 |
| Phase II | 47/783 (6.01%) | CAMI-DM 2.0 | 0.880 (0.843–0.912) | 0.325 (0.236–0.436) | 0.771 | 0.872 | 0.765 | 0.192 | 0.982 |
|  |  | CAMI-DM 1.0 | 0.815 (0.767–0.861) | 0.225 (0.160–0.327) | 0.750 | 0.787 | 0.747 | 0.166 | 0.974 |
|  |  | GRACE Score | 0.793 (0.734–0.854) | 0.225 (0.162–0.331) | 0.700 | 0.766 | 0.696 | 0.138 | 0.968 |

**# Newly recognized diabetes refers to patients with no prior diagnosis of diabetes, no history of diabetes, and no record of glucose-lowering therapy, who were newly diagnosed with diabetes at the time of admission or had HbA1c ≥ 6.5%. Previously diagnosed diabetes refers to patients with a documented history of diabetes or prior glucose-lowering therapy.**

**# Phase I includes patients enrolled between January 2013 and September 2014 with a diagnosis of STEMI or NSTEMI who were admitted within 7 days of symptom onset. Phase II includes patients enrolled between September 2014 and January 2016, with inclusion restricted to those hospitalized within 3 days of symptom onset.**
